# Functionally selective signaling and broad metabolic benefits by novel insulin receptor partial agonists

**DOI:** 10.1038/s41467-022-28561-9

**Published:** 2022-02-17

**Authors:** Margaret Wu, Ester Carballo-Jane, Haihong Zhou, Peter Zafian, Ge Dai, Mindy Liu, Julie Lao, Terri Kelly, Dan Shao, Judith Gorski, Dmitri Pissarnitski, Ahmet Kekec, Ying Chen, Stephen F. Previs, Giovanna Scapin, Yacob Gomez-Llorente, Scott A. Hollingsworth, Lin Yan, Danqing Feng, Pei Huo, Geoffrey Walford, Mark D. Erion, David E. Kelley, Songnian Lin, James Mu

**Affiliations:** 1 Merck & Co., Inc., Kenilworth, NJ 07033 USA; 2 Merck & Co., Inc., South San Francisco, CA 94080 USA; 3 Merck & Co., Inc., Boston, MA 02115 USA

**Keywords:** Type 2 diabetes, Receptor pharmacology

## Abstract

Insulin analogs have been developed to treat diabetes with focus primarily on improving the time action profile without affecting ligand-receptor interaction or functional selectivity. As a result, inherent liabilities (e.g. hypoglycemia) of injectable insulin continue to limit the true therapeutic potential of related agents. Insulin dimers were synthesized to investigate whether partial agonism of the insulin receptor (IR) tyrosine kinase is achievable, and to explore the potential for tissue-selective systemic insulin pharmacology. The insulin dimers induced distinct IR conformational changes compared to native monomeric insulin and substrate phosphorylation assays demonstrated partial agonism. Structurally distinct dimers with differences in conjugation sites and linkers were prepared to deliver desirable IR partial agonist (IRPA). Systemic infusions of a B29-B29 dimer in vivo revealed sharp differences compared to native insulin. Suppression of hepatic glucose production and lipolysis were like that attained with regular insulin, albeit with a distinctly shallower dose-response. In contrast, there was highly attenuated stimulation of glucose uptake into muscle. Mechanistic studies indicated that IRPAs exploit tissue differences in receptor density and have additional distinctions pertaining to drug clearance and distribution. The hepato-adipose selective action of IRPAs is a potentially safer approach for treatment of diabetes.

## Abstract (web summary)

Insulin therapies for patients with diabetes have challenges, including diminished hepatic preference of insulin action compared with endogenous insulin. Here the authors characterize insulin dimers that function as insulin receptor partial agonists, and exhibit hepatic and adipose tissue preference of insulin action and metabolic benefits in preclinical models.

## Introduction

Receptor tyrosine kinases (RTKs) mediate critical signaling events to regulate growth and metabolism. While functional selectivity between ligand and receptor interaction is common in nature, and evident in many classes of receptors with pleiotropic actions, biased signaling and partial agonism from a pharmacologic perspective of drug discovery has mostly been investigated in G protein-coupled receptor (GPCR)^1^. The topic has not been widely studied in the arena of RTK drug discovery though there are interesting cases of sub-pathway specific signaling for multiple RTK families^2^. Several examples are: (1) the seven epidermal growth factor receptor (EGFR) for which natural ligands can stabilize receptor dimers with distinct conformations, different levels of agonism and signaling bias^3^; (2) the fibroblast growth factor receptor (FGFR) family of four receptors that collectively have 18 ligands, a clear indication of a potential for signal fine-tuning^4^; and (3) insulin and insulin-like growth factor 1 (IGF-1) as respective “biased” ligands for the IGF-1 receptor (IGF-1R) or the insulin receptor (IR) to regulate a balance between growth and proliferation^2^. The physiological relevance of these native systems that manifest a potential for biased signaling is just beginning to be realized and the modulation by design of RTK ligands to harvest pharmacological benefit has yet to be broadly attempted^5^.

Insulin plays a fundamental role in metabolic homeostasis and its dysregulation underlies many metabolic disorders, including diabetes mellitus (DM) and cardiometabolic complications. For example, up to 68 percent of people age 65 or older with diabetes die from cardiovascular disease^6^. Recombinant insulin (or its analogs) is an essential therapy for patients with type 1 DM (T1DM) and many patients with type 2 DM (T2DM). However, challenges of current insulin therapies, including narrow therapeutic index (TI) to hypoglycemia and body weight gain, limit their adoption and, when adopted, challenge patients’ ability to achieve ideal glycemic control as a consequence of underdosing due to fear of hypoglycemia^7,8^.

Endogenously secreted insulin, by its entry into the portal circulation and with a considerable first-pass extraction by the liver (>50% first pass), establishes a physiological gradient of greater exposure for liver relative to peripheral tissues. The normal hierarchy of tissue insulin responsiveness: adipose > liver » skeletal muscle, together with the large extraction of endogenous insulin by the liver, interdigitates with the portal entry of insulin secretion to yield a fasting pattern of insulin action that is hepato-adipose preferential^9,10^. During postabsorptive conditions, control of hepatic glucose production (HGP) and rates of lipolysis, more than stimulation of glucose uptake by muscle and other tissues, are the fulcrum for control of fasting blood glucose, and indeed, most glucose utilization during fasting is insulin-independent, notably by the brain^10,11^. Yet, this pattern of healthy postabsorptive metabolism has been vexingly difficult to replicate with exogenously administered insulin. The portal-to-peripheral concentration gradient for exogenous insulin is abrogated by the systemic absorption of its subcutaneous injection depot, causing an equipoise of portal and peripheral exposure that undercuts a preferential hepatic locus of insulin action^11^.

Modern insulin analogs include rapid acting and basal insulins^12,13^, the former designed for relatively short-term control of rising glucose after meals and the latter, basal insulin, with an extended duration of action to regulate postabsorptive metabolism and control fasting glucose. Nearly all insulin-treated patients with diabetes utilize basal insulin, and while there are pharmacokinetic (PK) and potency differences across available basal insulins, all are full agonist insulin analogs, as is also true for the rapid acting insulin analogs. One of the most striking limitations of exogenously administered insulin is the risk of iatrogenic insulin-induced hypoglycemia, a risk recognized immediately after the advent of insulin therapy a century ago and which persists today despite an unrelenting effort to mitigate this risk, through frequent self-monitoring of blood glucose and informed adjustments of insulin dosage. Exogenous insulin therapy has a quite narrow TI, that is, a dose that can induce hypoglycemia is generally only slightly greater than a dose needed to achieve normal blood glucose^14^. The abrogation of the normal physiology of a preferential hepatic insulin action is one contributor to the risk of hypoglycemia with a basal insulin. A recent effort has explored a hepato-targeted insulin, characterized by a larger sized pegylated insulin^15^, the size of which exploited the fenestration of hepatic vascular sinusoids compared to tight-junction muscle capillary endothelium. In late-stage clinical studies^16^, this pegylated insulin reduced nocturnal hypoglycemia and weight gain, however, ultimately failed due to a lack of adequate control of lipolysis and triglyceride regulation^16,17^.

In the current studies, a novel class of insulin dimers is described that function as insulin receptor partial agonists (IRPA). The studies expand on an early observation^18^ that selected covalent dimers of insulin may have liver-preferential action and newly demonstrate that this can be tuned structurally to manifest desirable partial agonism across species. The partial agonism of IRPA derives from differences from insulin in IRPA binding to the insulin receptor, differences in receptor mediated ligand endocytosis, and selectivity or bias in downstream signal transduction. In vivo studies demonstrated clear differentiations from insulin in rodents and larger animal species of pig and dog, which derive mechanistically from the partial agonism and functional selectivity of signaling. Notably, these in vivo differences include a slower clearance than regular insulin, attributable largely to diminished IR mediated clearance, to a hepato-adipose tissue predominance in insulin action with relative sparing of skeletal muscle insulin action, and for a lesser propensity to cause hypoglycemia, a frequent complication of conventional insulin therapy. Taken together, these in vivo characteristics of IRPA form a profile that bears strong resemblance to that of the profile of postabsorptive physiology governed by endogenously secreted insulin in healthy humans.

## Results

### Selected insulin dimers function as IR partial agonists and induce biased signaling

**Fig. 1:**
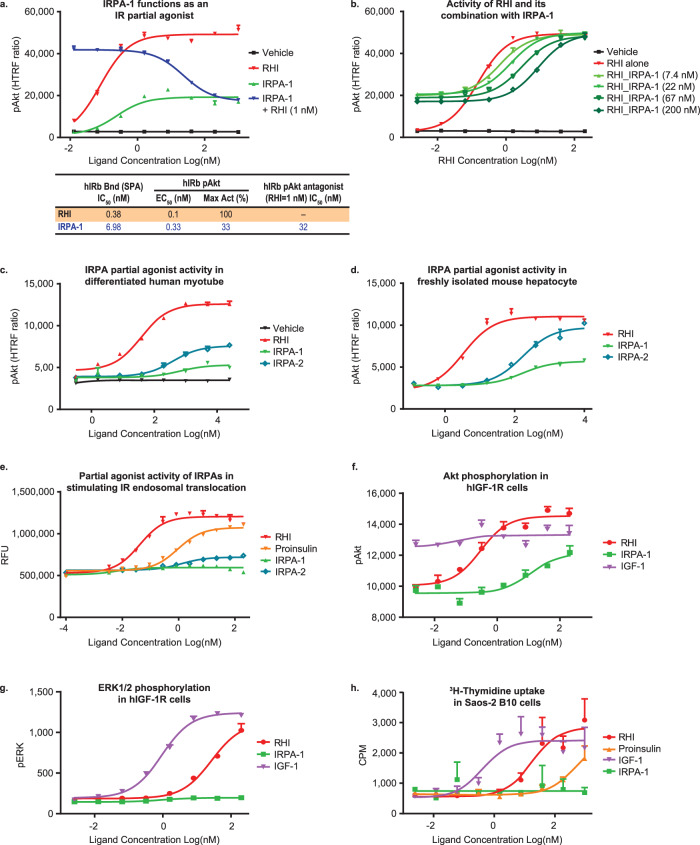
Selected covalent insulin dimers function as partial agonists and induce biased signaling. IRPA-1 functions as an IR partial agonist and is also a partial antagonist (**a**, **b**). Human IRb agonist activity (IRPA alone) or partial agonist activity (in the presence of 1 nM of RHI) of IRPA-1 in vitro in CHO-hIRb cells as measured by pAkt (**a**). RHI was also titrated alone or in the presence of fixed dose of IRPA-1 and the pAkt readouts are shown in **b**. IRPAs function as IR partial agonists in human myotube (**c**) and mouse hepatocyte (**d**). All titrations in a-d were conducted in duplicate (mean ± SD). IRPAs show reduced stimulation of IR internalization relative to RHI (**e**). The study was conducted using PathHunter Enzyme Fragment Complementation Assay (in quadruplicate, (mean ± SD)) to measure IR internalization (Endosome Translocation; DiscoverX). IRPA-1 induces differential pAkt vs. pERK signaling in HEK 293 cells expressing human IGF-1R (**f**, **g**) and has minimal stimulation of cellular proliferation (**h**). All titrations in f-h were conducted in triplicate (mean ± SD). Source data are provided as a Source Data file.

A series of IRPA molecules were generated to explore structure-function relationships within IRPA and with differing degrees of partial agonism, nine of these compounds were used in the current studies and their respective structures, potency, and degree of partial agonism are presented in Table 1. Compounds with similar functionality were used interchangeably in preclinical studies, an approach that serves to confirm class-specific properties rather than a compound-specific idiosyncratic effect. In general, IRPA molecules with higher potency and higher partial agonism were used in rodent studies due to the species’ relative insulin resistance. IRPA-1 is a novel insulin covalent dimer analog conjugated with a B29-B’29 linkage (see structure in Fig. S1a), shown to bind to the human insulin receptor isoform b (hIRb; table insert in Fig. 1a). IRPA-1 functions as a hIRb partial agonist when tested in CHO-hIRb cells and measuring IR-mediated phosphorylation of Akt (pAkt; Fig. 1a). Unless otherwise indicated, partial agonism is expressed as percentage of the maximum pAkt activity induced by RHI, this is due to the robustness of this assay and higher numerical values obtained as compared to IR phosphorylation (pIR). In this report, higher partial agonism denotes agonism that more closely approaches full agonism (i.e. a higher ratio for pAkt_IRPA_ to pAkt_RHI_), and the converse applies to lower partial agonism. That IRPA-1 behaves as a characteristic receptor partial agonist is illustrated by the attenuated maximum pAkt level when titrated alone (green line, Fig. 1a) and by its ability, at a high concentration of IRPA-1, to attenuate the action of RHI when combined (blue line, Fig. 1a; green and blue lines converge at high IRPA concentration). At low RHI exposure, IRPA-1 demonstrated additive activity but a partial antagonizing action with RHI at a high concentration, as determined when RHI was titrated upward in the presence of a fixed level of IRPA-1 (Fig. 1b). In contrast, a weak IR agonist (such as proinsulin) has a different profile from that of IRPA, revealing itself as a weak but full agonist and with a lack of antagonist activities under these conditions (Fig. S1b). In addition to partial agonist activity towards hIR, IRPA-1 demonstrated this property with IR of other species, including pig and dog (Fig. S1c, d). This cross-species similarity in partial agonism is also true in primary cells, e.g. differentiated human myotubes (Fig. 1c) and freshly isolated mouse hepatocytes (Fig. 1d). The partial agonism function was clearly structure based as a change of conjugation site (discussed below) or subtle modifications of the linker yielded changes in the level of partial agonism. For example, IRPA-2 compared to IRPA-1, the former without urea capping groups at the A1 and B1 positions (see Table 1 and Fig S1a for structural details), induced higher partial agonism (Fig. 1c, d and Fig. S1c, d). Another aspect of the IRPA compounds relative to RHI was a reduced maximum ligand-induced IR internalization. IRPA-1 has minimal activity to stimulate IR-internalization while IRPA-2 showed ~20% maximal activity in this assay (Fig. 1e).

**Table 1:** Structural information and in vitro data of IRPA dimers*.

Cmpd	Alternative name	In vitro assays (hIR)					
		Linker site	Linker	Capping group	IR Bind EC_50_ (nM)	pAkt EC_50_ (nM)	pAkt Max Act (%)
RHI		—	—	—	0.4	0.07	100
IRPA-1	MK-5160	B29-B29’	C8	NH_2_(CO)-	6.98	0.12	29
IRPA-2		B29-B29’	C8	None	1.34	0.12	43
IRPA-3		B29-B29’	C8	CH_3_(CO)-	2.02	0.13	26
IRPA-4		B3-B3’	Trz	None	0.22	0.006	51
IRPA-5		B29-B29’	Trz	None	1.09	0.11	37
IRPA-6		B1-B29’	Trz	None	0.41	0.04	92
IRPA-7	MK-1092	B29-B29’	cHex	None	2.60	0.14	43
IRPA-8		B29-B29’	Peg5	(CH_3_)_2_	1.28	0.03	27
IRPA-9		B1-B1’	Trz	None	0.53	0.01	96

^*^ Refer to Fig. S1A for the structures of linkers and capping groups. B3 N → K and B29 K → R mutated insulin was used for IRPA-4 (B3–B3′ dimer).

Since phosphorylation of different subdomains of the IR has been associated with different effector functions^19^, phosphorylation of key IR intracellular tyrosine residues was measured upon stimulation by representative IRPA molecules. Results in CHO-hIRb cells are summarized in Fig. S1e and indicate that the IR kinase domain tyrosine (i.e., Y1150) was preferentially phosphorylated by IRPAs relative to residues in the juxta-membrane region (i.e., Y960) or the carboxy-terminal region (i.e., Y1345). IR tyrosine phosphorylation at the juxta-membrane and carboxy-terminal regions are implicated in mediating IR-internalization and ligand-mediated recycling, while insulin-induced phosphorylation of the kinase domain is primarily responsible for propagation of downstream substrate phosphorylation^20^. Our interpretation is that the proportionately weaker phosphorylation of the juxta-membrane and carboxy-terminal regions correlates with reduced IRPA activity to stimulate IR-internalization.

In addition to differential IR tyrosine phosphorylation, IRPA also induces biased signaling among IR/IGF-1R’s downstream effector proteins. For example, in CHO-hIRb cells and HEK293 cells stably expressing human IGF-1R, IRPA-1 showed a partial agonist activity on pAkt relative to RHI (Fig. S2a; Fig. 1f), but a more blunted activity on ERK phosphorylation (pERK1/2) (Fig. S2b; Fig. 1g; compare pAkt vs. pERK activity of IRPA-1 in corresponding figures); in these studies, IGF-1 and RHI are fully active on both substrates. The blunting of ERK phosphorylation suggests less mitogenic potential for IRPA. To evaluate IRPA-1’s ability to stimulate thymidine uptake, Saos-2 cells were assessed, and minimal activity was detected. Under the same conditions, IGF-1, RHI and proinsulin each stimulates thymidine uptake, regardless of differing potency (Fig. 1h). This reduced mitogenic potential if IRPA correlates well with its low binding to IGF-1R (Fig. S2c).

### IRPAs induce distinct conformational changes and HDX signatures on IR-extracellular domain

**Fig. 2:**
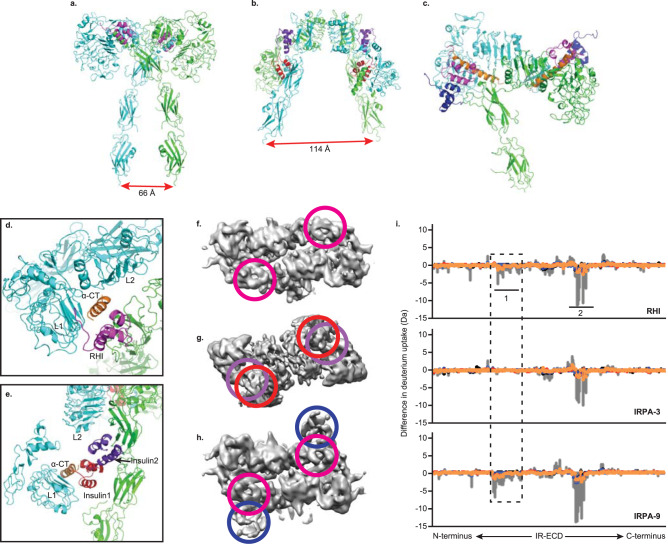
IRPAs induce distinct conformational changes and HDX signatures on IR-extracellular domain. Conformational changes of IR-ECD upon binding to RHI or IRPAs were evaluated using single particle cryo-EM and HDX-MS. Upper panel shows the ribbon representation of IR-ECD dimer: IR-ECD dimer complexed with two RHIs (**a**), two IRPA-3 partial agonists (**b**; PDB code 7MD4) and two IRPA-9 full agonists (**c**; PDB code 7MD5). Each IR-ECD monomer is colored in green or cyan. RHI or insulin from IRPAs are colored in magenta, red, purple or blue, respectively. The α-CT helix is colored in orange. Lower left panel shows zoom view of **a** and **b** to illustrate relative positions of L1, α-CT helix, L2 domain and the bound RHI or IRPA. Zoom view for RHI bound structure (**d**): RHI (magenta); and IRPA-3 bound structure (**e**): insulin1 (red), and insulin2 (purple). Lower middle panel are reconstructed maps for IR-ECD bound with RHIs, IRPA-3 and IPRA-9 (**f**–**h**), respectively. Colored circles represent RHI or one of the insulin monomers in the covalently linked insulin dimer molecules (IRPAs), which correspond to the ones shown in **a**–**c**. The same insulin in IRPAs is colored the same for illustration purpose. Lower right panel shows hydrogen/deuterium exchange difference plots for RHI and IRPAs (i). Region 1 consists of sequences in IR L2 domain: IRGGNN (344–349) and RSYAL (372–376), which differentiate IRPA partial agonist from full agonist binding (within the dashed line). Region 2 consists of sequences in IR α-CT helix: FRKTFEDYLHNVVF (701–714). Underlined amino acids were the interaction sites detected in cryo-EM studies. Deuterium uptake protection at region 1 was clearly detected for RHI and IRPA-9, both of which are IR full agonists. In contrast, no difference in deuterium uptake was seen for IRPA-3, an IR partial agonist.

To explore a structural basis by which the binding of IRPA to the IR might elucidate partial agonism and biased signaling pharmacology, single particle cryo-EM studies were conducted in the presence of RHI or IRPA to examine the structure of IR-extracellular domain (IR-ECD). The cryo-EM map for the IRPA-3 bound IR-ECD (Fig. 2b) was clearly different from the T-shape density observed for the RHI bound IR-ECD (Fig. 2a)^21^, and more closely resembled the inverted V shape in the negative staining analysis of the IR^22^ and to the crystallographic dimer^23^. Previous structural studies with RHI revealed the presumed fully activated conformation of IR in complex with either two insulin molecules^21^ or four insulin molecules^24^ which positioned the two “legs” of the receptor made up of the consecutive Fn-III domains in relative close proximity. In contrast to those fully activated receptors, the IRPA-3 complex, which acts as a partial agonist, engaged an α-CT helix and the L1 domain (IR site 1) with one insulin monomer and interacted with the Fn-III(I) domain (IR site 2 as defined earlier^24^) with the second insulin monomer. With the IRPA-3 study, the two “legs” of the receptor were much further apart than in a fully activated state (Fig. 2a, b), 114 Å versus 66Ǻ. Additionally, comparison between RHI and IRPA-3 bound structures around the α-CT helix region (Fig. 2d, e) revealed that although both RHI and IRPA-3 engaged IR site 1, the L1: insulin: α-CT assembly was in close contact with the L2 domain in the RHI bound structure, and away from the L2 domain in the IRPA-3 bound structure. These observations suggest that IRPA-3 binding prevents IR-ECD from transitioning to fully activated conformation.

Cryo-EM structure for a complex of a near-full agonist IRPA with IR-ECD (IRPA-9:IR-ECD) was also obtained (Fig. 2c). Indeed, the resulting IRPA-9 bound IR-ECD closely mimics the T-shape RHI bound structure where one insulin monomer is bound to the α-CT helix and the L1 domain, and the other exposed to the solvent with no direct interactions with the IR. For clarity, relative positions of insulin bound to IR-ECD are also depicted in the reconstructed maps using colored circles: IR-ECD bound with two RHIs (Fig. 2f), two IRPA-3 partial agonists (Fig. 2g) and two IRPA-9 full agonists (Fig. 2h).

To further understand the conformational changes in IR-ECD induced by RHI or IRPA binding, hydrogen-deuterium exchange mass-spectrometry (HDX-MS) was employed to measure differences in deuterium uptake between the unbound and ligand-bound states. H/D exchange difference plots are shown in Fig. 2i. Among the two regions that show pronounced H/D exchange protection upon ligand binding, region 2 around residues 701-714 (α−CT helix) showed protection across all three comparisons (RHI, IRPA-3 or IRPA-9 *vs*. unbound IR-ECD); protection at the IR α-CT region in response to insulin binding has been previously reported^25^. In contrast, region 1 spanning residues 344-349 and 372-376 (L2 domain) exhibited protection only in RHI and IRPA-9 (full agonist) bound IR-ECD but was largely diminished in the IRPA-3 (partial agonist) bound state. Having high resolution ligand-bound IR-ECD structures, H/D signatures can be clearly understood. In the RHI bound cryo-EM structure, one side of α−CT helix interacted with RHI and the L1 domain, and the other side engaged with the L2 domain (Fig. 2d). However, in the IRPA-3 bound structure, one insulin monomer did engage the α−CT helix, but the opposite side of the α−CT helix was away from the L2 domain, leaving L2 domain solvent exposed (Fig. 2e). Hence, H/D protection at region 2 (α-CT helix) was detected in all three HDX-MS comparisons but no protection was observed at region 1 (L2 domain) for IRPA-3 bound IR-ECD. In addition to the H/D signatures detected in region 1 and 2, many other relatively smaller H/D exchange protection or deprotection signatures were also observed for RHI or IRPA-9 bound (Table S1) and IRPA-3 bound IR-ECD (Table S2). Remarkably, many of the H/D signatures detected overlapped with the interaction sites observed in the cryo-EM maps (underlined sequences in Tables S1 and S2).

### IRPAs mediate attenuated signaling in muscle relative to liver

**Fig. 3:**
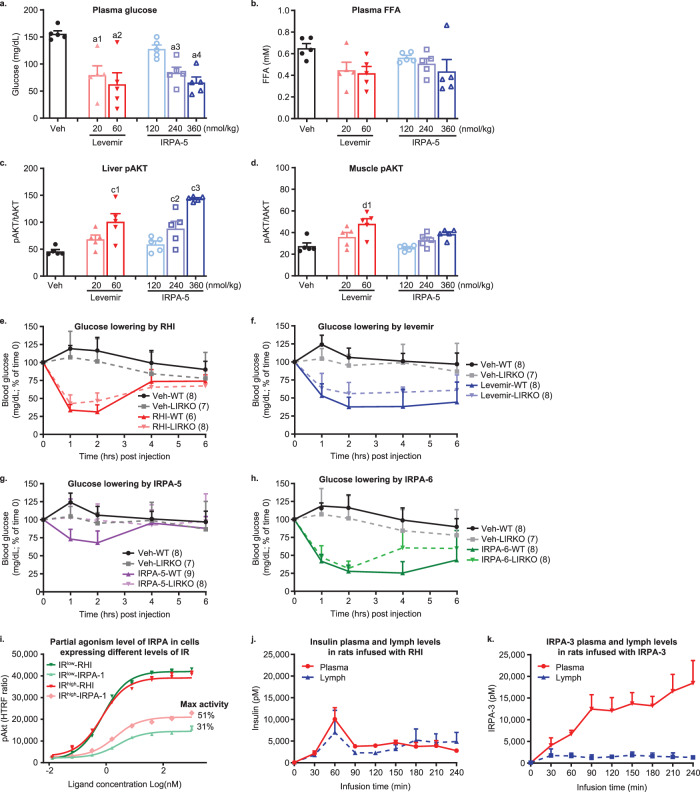
IRPAs induce differential tissue action compared to regular insulin. IRPAs have attenuated insulin-signaling in mouse muscle relative to their liver action (**a**–**d**). C57BL/6 mice were injected with insulin analogs followed by tissue collection 2 h post dose. Plasma glucose (**a**), free fatty acid (FFA; **b**), liver (**c**) and muscle pAkt/tAkt (**d**) were measured. *p* value (vs. vehicle) is calculated using one way ANOVA with Dunnett’s multiple comparisons test: a1 = 0.002, a2 = 0.0002, a3 = 0.003, a4 = 0.0003; c1 = 0.001, c2 = 0.01, c3 < 0.0001; d1 = 0.0005. *n* = 5 per group, mean ± SD. IRPAs show minimal glucose lowering activity in mice with liver insulin receptor deletion (**e**–**h**). RHI treated AAV-LIRKO mice had 30% AUC reduction compared to WT mice treated in the similar fashion (**e**). Corresponding AUC reduction for Levemir (**f**), IRPA-5 (**g**; partial agonist dimer) or IRPA-6 (**h**; high partial, close to full IR agonist dimer) were 41%, 102% or 43%, respectively. Dose of compounds are, RHI: 30 nmol/kg; Levemir: 24 nmol/kg; IRPA-5: 120 nmol/kg and IRPA-6: 60 nmol/kg. *N* number indicated on graph (legend in parathesis; mean ± SD. Dependency of partial agonism level of IRPA on IR receptor expression (**i**). CHO-hIR cells expressing two different levels of IR were generated. IR^high^ has 1.6x more membrane IR compared to IR^low^. IRPA-1 shows higher maximal activity in CHO-hIR cells expressing higher levels of IR, while RHI maximal activity is not impacted by IR density. Sample titration was conducted in duplicate (mean ± SD). IRPAs have attenuated peripheral distribution (**j**, **k**). Plasma and lymph partition in rats infused with RHI (*n* = 5; mean ± SEM) or IRPA3 (*n* = 4; mean ± SEM) were compared. There was no significant difference between plasma and lymph RHI levels. *p* = 0.0017 for IRPA-3 plasma and lymph levels, two-way mixed effects ANOVA with Geisser-Greenhouse correction. Source data are provided as a Source Data file.

IRPAs can lower blood glucose in mice as effectively as conventional insulins, albeit a higher dose of IRPA is often needed to yield similar efficacy. For example, a single dose of IRPA-5 dose-dependently lowered glucose 2-hours post dosing in normal C57BL/6 mice, achieving similar efficacy as Levemir (a lipidated long-acting insulin) (Fig. 3a). Also, demonstrating target engagement in adipose (see Fig. S4a for IRPA-5-mediated glucose uptake in differentiated adipocyte in a partial agonist fashion) as well as in the liver, IRPA-5 and Levemir reduced free fatty acid (FFA) similarly (Fig. 3b). Tissue pAkt relative to total Akt level (pAkt/tAkt) was measured to provide an index of insulin signal transduction activation and a dose-dependent increase for pAkt in liver was seen with both Levemir and IRPA-5 (Fig. 3c). In contrast, a similar analysis in skeletal muscle pAkt revealed attenuated signaling induced by IRPA-5 compared to Levemir (Fig. 3d), and of note, with a low variability of the IRPA-5-mediated muscle Akt activation (Fig. 3d). Due to adipocyte’s higher sensitivity to insulin, adipose tissue pAkt appears to be saturated even at the lowest dose of insulin tested under the same condition (Fig. S4b).

To further assess IRPA’s tissue-specific action, AAV8-cre was introduced into IR conditional knockout (cKO) mice to create liver-specific IR deletion mice (named AAV-LIRKO to distinguish with the original LIRKO mice described earlier^26^). AAV-cre-mediated liver IR deletion combined with a high fat diet (HFD) induced severely insulin resistant diabetic phenotype manifesting hyperglycemia and hyperinsulinemia (Fig. S5a, b). A single administration of RHI or an IRPA molecule in AAV-LIRKO mice allowed assessment of glycemic efficacy and its dependency on liver insulin action. IR full agonists showed a mild reduction of glycemic efficacy in mice lacking the liver insulin receptor; for example, RHI and Levemir had comparable reductions in glucose lowering efficacy in AAV-LIRKO mice compared to wild type (WT) mice, 30% and 41% for RHI (Fig. 3e) and Levemir (Fig. 3f), respectively. Slightly higher dependency on liver IR by Levemir (vs. RHI) probably reflects its weak liver-preferring action as observed in human^27^. IRPA-5, albeit less potent than RHI, lowered glucose in WT mice but the glucose lowering action of IRPA-5 was completely lost in AAV-LIRKO mice (Fig. 3g; 100% loss of action). In contrast, IRPA-6, an insulin dimer with 92% maximum pAkt activity, behaved similar as Levemir in AAV-LIRKO mice (Fig. 3h; 43% reduction in glucose AUC relative to its efficacy in WT mice). Collectively, these results in AAV-LIRKO mice support the concept that insulin dimers like IRPA-5 have a liver-preferential action. On the other hand, IR full agonists (e.g. RHI) as well as insulin dimers that have high partial agonist activities (e.g. IRPA-6), maintained effective glucose lowering capability in AAV-LIRKO mice, likely due to compensatory stimulation of glucose uptake in non-hepatic tissues.

It has been well-documented in the GPCR field that the degree of partial agonism for a given partial agonist ligand is additionally modulated by the cellular density of its receptor^28^, i.e., a full agonist has the same maximum effect (at saturating concentrations) in cells having different receptor expression while a partial agonist induced smaller maximum effect in low expressing cells relative to cells having high receptor expression^28,29^. This unique property of a GPCR partial agonism forms the basis for an effective approach when differential tissue action is desirable for pharmacological targeting. Accordingly, we postulated that the attenuated action of an IRPA observed in skeletal muscle could be due to lower IR expression in muscle. Higher liver than muscle IR expression has been documented^30^ including in our own studies^26^. To formally evaluate the dependency of IRPA partial agonism level on IR expression, two CHO cell lines were generated that expressed different levels of hIR (by 60% based on IR in the membrane fraction) and though both lines reached a same maximal pAkt activation upon RHI stimulation, IRPA-1 treatment yielded higher maximal activity in the higher IR-expressing CHO cells (51% and 31% for IR^high^ and IR^low^ cells, respectively; Fig. 3i). These results provide one plausible factor underlying a stronger liver relative to muscle action for IRPA. Thus, analogous to GPCR partial agonists, IRPAs possess an intrinsic property to exploits physiological differences in IR expression of different tissues and thereby shape an in vivo pharmacology profile that manifests tissue selectivity.

Difference between liver and muscle in IRPA delivery is another consideration because, in contrast to the highly fenestrated endothelium of the liver which enables rapid and full access of circulating insulin, skeletal muscle has capillaries with tight vascular endothelial junctions, for which IR-mediated insulin transport governs delivery^31^. Lymphatic or interstitial insulin levels have stronger correlation with muscle glucose disposal than plasma insulin^32^, indicating that endothelium transcytosis of insulin is a rate-limiting step in insulin stimulation of muscle glucose utilization. Since IRPAs have reduced cellular internalization (Fig. 1e), we considered whether reduced transcytosis by IRPA molecules may also contribute to an attenuated muscle IR activation. Plasma and lymphatic drug levels were measured during RHI or IRPA infusion in rats, and as shown in Fig. 3j, k, RHI infusion led to comparable lymph and plasma drug levels while in contrast, infusion of the same dose of IRPA-3 resulted in a low lymphatic drug concentration despite a much higher plasma concentration. Similar study of a high partial agonist, IRPA-6, yielded a profile close to that of RHI (Fig. S4c) indicating partial agonism level of an IRPA is the main driver for differential transcytosis. In summary, the observed attenuated IRPA action in muscle relative to liver can be attributed to a low IR expression in muscle and reduced IRPA endothelium transcytosis and delivery to muscle.

### IRPAs demonstrate hepato-adipose preferential action in dog

**Fig. 4:**
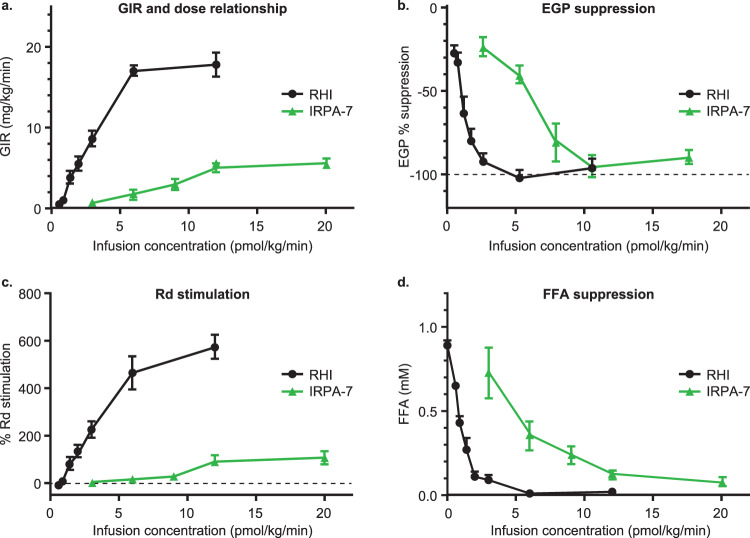
IRPAs have hepato-adipose preferential action in higher species. Differential dose-dependent GIR of IRPA-7 vs. RHI under euglycemic dog clamp (**a**–**d**). A euglycemic–hyperinsulinemia clamp (glucose at 100 mg/dL) in the beagle dogs was employed to study differences in steady-state GIR between IRPA-7 and RHI under constant i.v. infusion. The studies followed a cross over design using the same dogs. IRPA-7 euglycemic dog clamp studies demonstrated attenuated stimulation of whole body (**a**) and muscle glucose metabolism (**c**) with effective suppression of hepatic glucose production (**b**) and adipose lipolysis (**d**). All data is presented as mean ± SEM. *n* = 5–8 animals/dose group (IRPA-7), *n* = 3–8 animals/dose group (RHI). Dose group with corresponding sample size in parathesis is RHI at 0.6 (7), 0.9 (8), 1.4 (8), 2 (8), 3 (8), 6 (3), 12 (8) pmol/kg/min; IRPA-7 at 3 (6), 6 (5), 9 (6), 12 (8) and 20 (6) pmol/kg/min. Source data are provided as a Source Data file.

To determine whether IRPA could assert a clear gradient of hepatic and adipose insulin action relative to that in skeletal muscle and as compared to RHI, euglycemic, pancreatic glucose clamp studies were conducted in beagle dogs. Rates of glucose infusion (GIR) were recorded and separate, sequential clamp studies (using the same cohorts of animals for a within-subject study design) with progressively higher infusion rates of IRPA (pmol/kg/min; pkm) were conducted. C-peptide determinations (data not shown) demonstrated complete inhibition of endogenous insulin secretion during the clamps. Measured plasma glucagon was 32 ± 1 pg/ml, with peripheral replacement. Using the protocol described in Fig. S6a, IRPA-1 elicited a gradual pharmacodynamic (PD) increase in GIR as infusion rates were increased (Fig. S6b). This contrasted with RHI, which demonstrated its previously characterized steep dose response for GIR. It has been empirically recognized that during clamp conditions of a relatively low GIR (i.e. 5 mg/kg/min and below in dogs) the principal sites of action of insulin is suppression of HGP and lipolysis^33^, but when GIR rises above 5 mg/kg/min and beyond, this reflects an increasing stimulation of glucose uptake into skeletal muscle. Across the range of infusion rates of IRPA, GIR remained in the lower range and to more precisely quantify tissue-specific actions, a non-radioactive (stable isotope) glucose tracer was introduced to the clamp studies allowing measurement of suppression of HGP and of glucose rate of disappearance (Rd). These tracer studies confirmed a liver-preferential action of IRPAs. For example, IRPA-7 (see Table 1 and Fig S1a for structure), a lead molecule that was later advanced to human studies (MK-1092)^34,35^, showed a gradual GIR increase in dogs as its dose escalated (Fig. 4a) and with complete HGP suppression (Fig. 4b), albeit at higher doses than RHI. Yet, even at the top dose of IRPA-7, there was only weak stimulation of Rd (Fig. 4c). Also, of note, IRPA-7 (Fig. 4d) could effectively suppress lipolysis, as indicated by the low plasma FFA levels. Collectively, these canine clamp studies, like the rodent studies, demonstrate that IRPA has a hepato-adipose preferential action, more closely resembling the gradient of postabsorptive metabolism associated with the actions of endogenously secreted insulin.

### IRPAs exhibit improved therapeutic index in glucose lowering in rodent

**Fig. 5:**
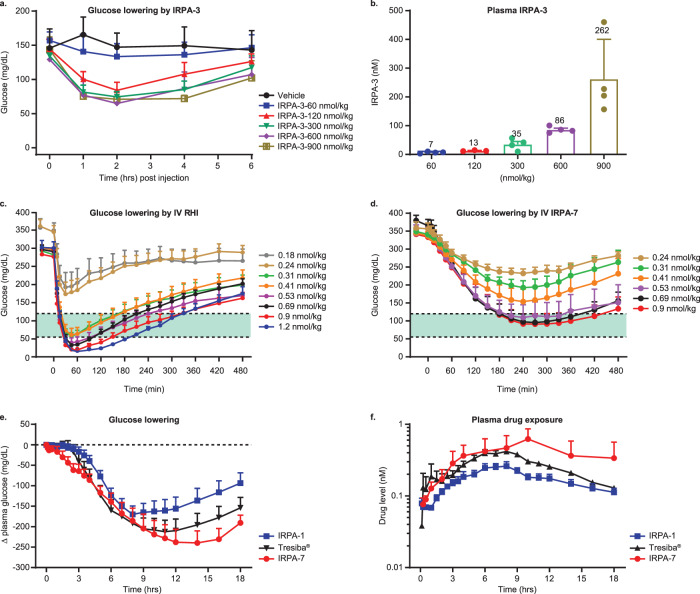
IRPAs have improved therapeutic index in glucose lowering. IRPA-3 lowers glucose effectively in acute testing with reduced hypoglycemic risk in mice (**a**; mean ± SD, *n* = 8). Plasma IRPA-3 measured at 2-h post dose was shown in (**b**; mean ± SD, *n* = 3 or 4). Acute glucose lowering by i.v. RHI (**c**) and IRPA-7 (**d**) in diabetic minipigs with dose escalation of 30% upwards. Improved therapeutic index of IRPA-7 is indicated by smooth titration of glucose lowering. Data is presented as mean ± SEM, *n* = 6 animals per dose group. IRPAs demonstrated protracted s.c. profile consistent with QD dosing in diabetic minipig (**e**, **f**). IRPA-1 and IRPA-7 dosed as single s.c. bolus yielded overall comparable PD profile relative to Tresiba® in diabetic minipigs. Plasma exposures for IRPA-1 and IRPA-7 were not different from those of Tresiba®. Data is presented as mean ± SEM, *n* = 20 animals for IRPA-1, *n* = 6 animals for IRPA-7 and *n* = 19 animals for Tresiba®. IRPA-7 was formulated in Glycerin, 16 mg/mL; Metacresol, 1.6 mg/mL; Phenol, 0.65 mg/mL; Anhydrous Sodium Phosphate, Dibasic, 7 mM; 0.671 equivalent Zn^2+^; pH 7.4. IRPA-1, IRPA-7 or Tresiba® dose used was 0.9, 0.9 or 0.35 nmol/kg, respectively. Source data are provided as a Source Data file.

Broadly, the therapeutic index (TI) for a medication is regarded as that increment of dose above a fully efficacious dose that can be safely tolerated before dose-related adverse effects ensue, and for insulin the TI is generally framed with respect to induction of insulin-induced hypoglycemia and it is notoriously narrow. When IRPA-3 was administered to C57BL/6 mice to lower glucose, the initial increases of dose increased efficacy (Fig. 5a; 60 vs. 120 nmol/kg), however, as the dose was further increased, little or no further increase of glucose lowering was detected (Fig. 5a; 300–900 nmol/kg). Interestingly, during this upward dose titration study, the plateauing of maximal glucose lowering hovered above the hypoglycemic threshold, denoting a widening of TI. Strikingly, this plateau of glycemia held as the circulating IRPA-3 level increased more than 20-fold (120 vs. 900 nmol/kg doses) (Fig. 5b). A similar amplitude of dose escalation of RHI is not possible without driving animals to severe hypoglycemia and even death^36^. The leveling off in glucose reduction regardless of dose escalation as blood glucose approaches normoglycemia denotes an improved TI for IRPA relative to conventional insulins and appears to be a phenomenon unique to partial agonist dimers since insulin dimers with full agonist activities do not have this characteristic (Fig. S7a–f). This apparent reduction in action at higher doses of IRPAs is not due to an emergent loss of intrinsic efficacy but rather suggests a plateau of its action and perhaps some manner of dependence upon the hyperglycemic condition for its action. Regarding this last point, doses of IRPA-1 which were essentially inactive to reduce normal levels of fasting glucose in non-diabetic mice, demonstrated potent glycemic efficacy in diabetic animals (Fig. S7a–c).

### Efficacy of IRPA in diabetic minipigs

Glucose lowering efficacy of IRPAs was also studied in Yucatan diabetic minipigs (alloxan induced severe insulinopenia with glucose levels ~350 mg/dL; Sinclair Research Center, MO^37^). Single dose escalation studies, administered intravenously to avoid confounding effects of differing subcutaneous absorption, revealed a different profile for IRPA compared to other insulin products. For example, in the diabetic minipigs, it proved difficult to upward titrate doses of RHI (Fig. 5c) or Lantus® (Fig. S7g) because as target normoglycemia was approached small increases precipitated hypoglycemia (the hypoglycemic threshold in minipigs is ~55 mg/dL). However, with IRPA, gradual escalation of dose up to and beyond achieving a target range of normoglycemia was feasible without inducing hypoglycemia, (Fig. 5d and Fig. S7h), denoting an improved TI for IRPA. In these minipig studies, IRPA shows slower clearance relative to RHI. For example, when both molecules were administered at the same i.v. bolus dose of 0.4 nmol/kg, mean clearance was 3.3 or 8.2 mL/min/kg for IRPA-7 or RHI, respectively. In addition to its intrinsic property of liver preferential action, slower clearance of IRPA can contribute to reduced hypoglycemic risk by allowing more time for animals to counter-regulate steep glucose lowering mediated by other insulins.

Similar skin architecture to humans makes the minipig a preferred preclinical species for evaluating insulin time action profiles following subcutaneous administration. Diabetic minipig studies of IRPA-1 and IRPA-7 formulated with Zn^2+^ (see Fig. 5e legend) demonstrated a PK and PD profiles that were not significantly different from Tresiba®, a lipidated insulin with a flat PK/PD profile^38^ (Figs. 5e, f for PD and PK respectively). The bioavailability of IRPA-1 after subcutaneous injection is ~60%. Compared to IRPA-1, IRPA-7 achieved higher drug exposure and glucose lowering efficacy together with a flatter PK profile, PK/PD differences that likely derive from the latter’s higher in vitro potency and partial agonism (Table 1). In addition to the attractive efficacy and time action profile, IRPA-1 and IRPA-7 have sufficient chemical stability and lack of neutralizing anti-drug antibody (ADA) formation after chronic dosing in rat or minipigs. Collectively, these results support further IRPA development in the clinic.

### Metabolic benefits of IRPA beyond glycemic control

**Fig. 6:**
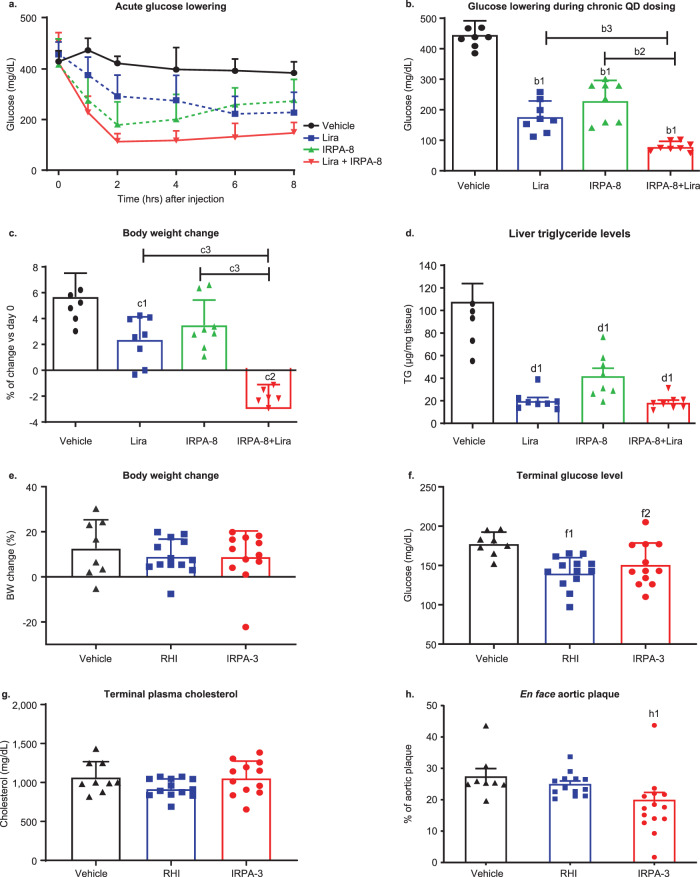
Beneficial IRPA effects beyond glycemic control. Glucose, body weight and hepatic triglyceride (TG) lowering by IRPA alone or in combination with a GLP-1 analog in mouse studies (**a**–**d**). Glucose lowering acutely (**a**) or during chronic QD dosing (**b**; 2-h post dose on day 11) in diabetic mice. Body weight (**c**) and liver triglyceride (**d**) changes at the end of 14-day QD dosing. Lira and IRPA-8 dose used was 3 or 18 nmol/kg, respectively. Data is presented as mean ± SD. *N* = 8. *p* value is calculated using one-way ANOVA with Tukey’s multiple comparisons test: b1 < 0.001 vs. vehicle, b2 < 0.001 and b3 = 0.002 for indicated comparisons; c1 = 0.008 and c2 = 0.0001 vs. vehicle, c3 < 0.0001 for indicated comparisons; d1 < 0.001 vs. vehicle. IRPA reduces aortic plaque in ApoE KO mice (**e**–**h**). There was no significant body weight or cholesterol change from baseline by RHI or IRPA-3 (**e**, **g**). Terminal glucose level in RHI and IRPA-3 group was significantly lower than vehicle group (**f**) and aortic plaque (**h**) level was significantly reduced in IRPA-3 group compared to RHI. Data is presented as mean ± SD. *p* value is calculated using one-way ANOVA with Tukey’s multiple comparisons test: f1 = 0.0034 and f2 = 0.035 vs. vehicle; h1 = 0.046 vs. vehicle. Sample size is 8, 13 or 12 for vehicle, RHI or IRPA-3, respectively. RHI or IRPA-3 was administered at 48 or 967 nmol/kg/day, respectively. Source data are provided as a Source Data file.

Chronic insulin therapy reverses the catabolic weight loss of poorly controlled diabetes and beyond this, often induces undesirable weight gain, aggravating risk of obesity. Persistent hyperinsulinemia is regarded as a causative factor and there is evidence that restoring the hepatic to peripheral gradient for insulin exposure and insulin action may mitigate risk of excessive weight gain. For example, clinical studies of Peglispro, a pegylated basal insulin with an hepato-preferential action, demonstrated less weight gain and in some individuals, small weight reduction^16,39^. IRPA’s effect on weight was studied in high fat diet (HFD)-STZ mice, a diabetic model of obesity, insulin resistance and compromised β-cell function^40^. During a 14-day study, IRPA-8, Liraglutide (a lipidated GLP-1 analog; “lira”) and the combination were administered daily via subcutaneous injection. As shown in Fig. 6a, b, IRPA-8 and lira had comparable glucose efficacy, acutely and during chronic treatment, while the combination showed added benefit. At study end, lira-treated mice had significantly less weight gain and intriguingly, weight gain in the IRPA-8 treated group also trended lower; impressively, the combination of the two treatments yielded a significant reduction of weight (Fig. 6c). Analysis of terminal liver triglyceride levels showed all three treatments resulted in a dramatic reduction of liver fat (Fig. 6d). Collectively, these results indicate IRPA, unlike full agonist insulins, may have a potential to mitigate risk of excessive weight gain.

Persistent systemic hyperinsulinemia is also regarded as a cardiovascular risk factor and in part, this risk is ascribed to the arm of insulin signal transduction that mediates mitogenic actions. Insulin receptor agonism with biased signaling towards lower ERK activation (relative to Akt pathway) has been proposed to have anti-inflammatory action in vascular endothelial cells^41^, and blunted ERK activation by IRPA was described above. To test whether IRPA has favorable action on aortic atherosclerotic plaque, IRPA-3 or RHI was administered for 8 weeks (infused via subcutaneous pump) in ApoE KO mice. Mice were pre-conditioned with a 1.25% cholesterol diet for 4 weeks before being maintained on a 0.5% cholesterol diet during the treatment phase. Terminal weight and glucose data are shown in Fig. 6e–f, and treatment did not affect plasma cholesterol (Fig. 6g). Compared to RHI treatment, IRPA-3 treatment led to a significant reduction in aortic plaque size (Fig. 6h), measured by Sudan IV staining after en face aorta dissection.

## Discussion

The current studies present an extensive set of data combining in vitro characterizations, structural biology mapping of ligand-receptor binding, preclinical in vivo murine and rodent pharmacology as well as in two large animal species (dog and minipig), to investigate a novel series of covalently linked insulin dimers. Selected molecules amongst these linked insulin dimers were shown to function as partial agonists of the insulin receptor and evoke a pattern of pharmacology that closely replicates the role of endogenously secreted insulin in the governance of postabsorptive glucose homeostasis. In the discussion of these novel findings that will follow, it is first worthwhile to succinctly outline the challenges posed for a basal insulin if it is to replicate normal physiology in those who require such therapy. In health, insulin secretion, released into portal circulation and with a high first pass extraction by the liver, exerts a hepato-selective action to govern the rate of glucose production by the liver, the principal site of fasting blood glucose control^10,11^. Together with the heightened insulin responsivity of adipose tissue relative to skeletal muscle, the low fasting levels of peripherally circulating insulin are sufficient to control the release of fatty acids, limiting this to a minute fraction of the considerable stores of adipose triglyceride. These actions occur without evoking much stimulation of glucose uptake into muscle, and thereby sparing circulating glucose for requisite insulin-independent consumption by the central nervous system. At first glance, given the severely insulin deficient condition of T1DM (and for many with advanced severity of T2DM), it might seem a simple issue of hormone replacement. Yet empirically, to achieve a precise replacement of insulin is anything but simple. The subcutaneous administration of exogenous insulin, absorbed into peripheral venous circulation, obliterates the normal portal to peripheral gradient of insulin concentrations and in so doing introduces an imbalance of insulin action. The consequence, recognized since the advent of insulin therapy a century ago, is that this exacerbates the risk for hypoglycemia^7^, and substantially defines the narrow TI of insulin and the disproportionate elevation of systemic insulin^11^. Multiple innovations in the design of basal insulin have focused upon smoothing and flattening the PK profile, worthy achievements but not transformative in addressing the above challenges.

Prior innovations seeking a transformative re-establishment of a hepato-preferential locus of action for exogenous basal insulin have entailed invasive peritoneal delivery^42^, fraught with catheter related complications, or enlargement of insulin via pegylation to exploit the unique fenestrated capillaries of the liver, an approach that markedly diminished delivery to adipose tissues and failed to adequately control lipolysis^15,17,43^. Our interest in exploring a potential role of insulin dimers was inspired by a provocative yet long overlooked report of canine investigations that indicated a hepato-preferential pharmacology^18^. A novel series of covalently linked insulin dimers were generated, and in vitro assays yielded the exciting findings that a subset of these can act as insulin receptor partial agonists. Structure-function interrogations revealed that partial agonism can be tuned by site of attachment of the linker (e.g. B29-B29 vs. B1-B1 linkage), and further tuned by subtle end-capping of insulin. Mapping of the sites of phosphorylation of the insulin receptor evoked by IRPA revealed that it differed significantly from that of native insulin. Furthermore, cryo-EM and HDX-MS analysis indicate that the presence of the second insulin monomer in IRPA prevents the receptor from being fully activated. The IRPA bound structure likely captured the intermediate state between the inactive and fully activated receptor. It is worth noting that single particle cryo-EM provides an averaged 3D image of IR-ECD: ligand complex. In contrast, HDX-MS measures the alteration in solution dynamics of IR-ECD induced by ligand binding. The high degree of consistency between these two orthogonal methods builds confidence in molecular characterizations of IR-ECD complexed with insulin and its analogs and bolsters the concept that partial agonism observed with IRPA derives from differences in its binding pattern to the IR. Collectively, these results provide novel insight of insulin receptor structure – function relationship, which forms the basis of better understanding of signaling bias and improved design of next generation therapies.

Partial agonists exist in nature and have been well explored in the drug discovery of GPCR pharmacology, yet much less so for receptor tyrosine kinases (RTK)^44,45^. Thus, the IRPA reported herein represents a major advancement, one of considerable importance for basic research in elucidating and parsing insulin signal transduction, and an innovation of pragmatic potential for therapeutic application, the latter being the chief focus of the current study. One aspect of IRPA modulation of insulin signal transduction discovered in the current study was a bias in downstream activation, less evocation of ERK activation relative to Akt phosphorylation triggering substrate metabolism (which was also seen in selected lipidated insulins^46^). Our preliminary studies suggest that a relative diminution of ERK activation by IRPA could mitigate risks of atherosclerosis^41^ and mitogenesis^47^ otherwise potentiated by sustained hyperinsulinemia, an observation requiring deeper investigation given its apparent clinical importance.

A central aspect of receptor partial agonists is exploiting physiological differences amongst cells and tissues in receptor density; this imbues a potential for a degree of tissue selectivity in ligand-receptor mediated actions. As cited above, developing novel approaches to a basal insulin that delivers hepato-preferential actions has been an elusive aspiration of exogenous insulin therapy. In this report, cellular studies in which the expression of the insulin receptor was engineered to be high versus low substantiated that IRPA action (i.e. maximum efficacy as exemplified by difference in respective pAkt plateau) follows the expression level of the insulin receptor, while a full agonist to IR (i.e. RHI) leads to the same degree of insulin pathway activation. Similarly, insulin receptor density difference, higher in liver and adipose than in skeletal muscle, results in stronger IRPA action in liver relative to muscle and contributes strongly to the hepato-preferential and to muscle-sparing actions of IRPA. In vivo rodent studies with conditional knock-down of the liver insulin receptor confirmed that liver is a critical locus of IRPA pharmacology in governance of glycemia. And in vivo canine glucose clamp studies strongly affirmed that it is the control of hepatic glucose production that is the principal site of action for IRPA. Thus, IRPA provides a novel, non-invasive means to preferentially target insulin action in the liver. The outcomes of the single ascending dose studies in human^34,35^ are in a manner concordant with observations in preclinical species, even though further studies are needed to fully compare IRPA to currently approved basal insulins and to evaluate its potential for reduced risk of hypoglycemia.

There is another aspect of IRPA action that is both unique and of considerable importance and this concerns the reduced insulin receptor mediated endocytosis of IRPA in muscle compared to native insulin. In normal physiology, a considerable proportion of receptor bound insulin undergoes endocytosis and intra-cellular degradation^20^, a pathway that accounts for a substantial proportion of insulin clearance from circulation. The in vitro insulin receptor phosphorylation mapping (after engagement with IRPA) revealed diminished activation of the juxta-membrane and carboxy-terminal tyrosines that mediate signaling for endocytosis of insulin. This has implications for systemic PK of IRPA, prolonged half-life relative to the short half-life of insulin. And it pertains directly to tissue selectivity of IRPA action, most notably with respect to skeletal muscle. As earlier noted, skeletal muscle is perfused by capillaries with tight endothelial junctions, anatomically quite different from fenestrated capillaries in hepatic sinusoids, and insulin delivery to the tissue bed of muscle is dependent upon insulin transcytosis^31^. Lymphatic catherization studies revealed greatly diminished delivery of IRPA (which appears to have an inverse relationship to its partial agonism level, supporting the observation IRPAs with 25-50% partial agonism reported here have distinctly attenuated muscle access) versus insulin into skeletal muscle, thus despite high circulating availability of IRPA, muscle exposure was selectively blocked. The canine clamp studies affirmed minimal stimulation of glucose uptake by muscle despite robust IRPA action in the suppression of hepatic glucose production.

It was of potential concern however that this phenomenon of diminished IRPA delivery might also preclude an adequate governance of lipolysis, arguably the key downfall of the pharmacology of pegylated insulin previously highlighted and that otherwise successfully controlled hepatic glucose production^17,39^. Yet, the in vivo rodent experiments and in-depth canine clamp studies demonstrated that concomitant with hepatic glucose production regulation, IRPA exerts control (i.e. suppression) of adipose lipolysis and consequent regulation of circulating levels of fatty acids. In healthy postabsorptive metabolism it is not only control of rates of glucose production but also control of rates of lipolysis that is governed by insulin; untoward effects of unrestrained release of fatty acids seriously perturb glucose homeostasis and set the conditions for lipotoxicity in non-adipose tissue, considered a strong basis for insulin resistance and organ damage. Adipose tissue responsivity to insulin is characteristically considerably higher than that of muscle, and at least equal or greater than liver, and we interpret the present finding of IRPA pharmacology in control of lipolysis to reflect this underlying tissue-based property of adipose.

Taken together, the in vivo actions of IRPA, to exert control of hepatic glucose production, to do this as well in adipose, and to largely preclude skeletal muscle begin to closely approximate the idealized profile of an exogenously administered basal insulin seeking to assert a normative pattern of postabsorptive metabolism. This pharmacology profile is important to the TI of IRPA compared to insulin. Endogenously secreted insulin very rarely causes hypoglycemia (apart from instance of untoward pharmacologic stimulation) due to tight negative feedback of blood glucose and with a short half-life of insulin. Exogenous administration of insulin has a notoriously narrow therapeutic index. The separation between a dose that achieves normoglycemia and one that induces hypoglycemia is a fine line, one that practitioners prescribing insulin doses and those who take insulin are vexed with trying not to cross on a daily, on-going basis^36,48^. In contrast to the steep dose-response to insulin, the dose-response for IRPA was decidedly more gradual. A practical implication is that a practitioner could conceivably advance a dose of IRPA to achieve control of fasting blood glucose with less immediate concern that each increment, even if slight, might tip the balance toward hypoglycemia. This is of course a postulate that will ultimately require more extensive clinical investigation. In addition to TI improvement in glycemic control, our studies reveal other aspects of IRPA pharmacology with potential to provide additional benefits compared to insulin, including less body weight gain and atherosclerotic lesion reduction, both of which are highly desirable for diabetic patients with comorbidities of obesity and / or dyslipidemia.

In summary, the discovery of a series of insulin receptor partial agonists opens a line of inquiry into the engineering of an exogenous insulin therapeutic that can non-invasively achieve a hepato-adipose preferential action, and be muscle sparing in its actions, and thereby achieve a close physiological replication of endogenously secreted insulin in its hierarchy of control of postabsorptive metabolism. These molecules can be excellent reagents to pry open the door more fully for a deeper understanding as to the mechanisms by which the insulin receptor tyrosine kinase separates and segregates aspects of its diverse pattern of signal transduction. IRPAs are potentially transformative agents for advancing our understanding of the hormone that has been a key medical discovery and advancement, now a century old.

## Methods

### Materials and chemicals

RHI (recombinant human insulin) was purchased from commercial sources. Earlier covalently linked dimeric insulin species were reported in the literature^49^. MRL medicinal chemistry effort on the SAR study of the dimeric insulin has resulted in the discovery of a novel series of compounds functioning as partial agonists of insulin receptor. IRPA-1 (MK-5160) is a novel B29-B’29 insulin dimer, which is connected via a suberic linkage, and A1 and B1 sites of each unit are substituted with urea groups (Table 1, Fig. S1A). Novel IRPAs linked with a triazole-based linkage, IRPA-4, 5, 6, and 9 were obtained via click chemistry. Each of these compounds was linked at different site, B3 to B3’, B29 to B1’, B29 to B29’, and B1 to B1’, respectively, for IRPA-4, 5, 6, and 9. IRPA-7 (MK-1092) is a B29-B29’ dimeric insulin linked with a conformational constrained trans-cyclohexane-1,4-diacid linker, and IRPA-8 is a B29-B29’ dimeric insulin linked with a short PEG based linker and all of its A1 and B1 positions were modified with dimethylation. The detailed structures and synthesis of these IRPA compounds can be found in Supplementary Material and Method Section.

### IRPA in vitro potency and selectivity

The potency and activity of IRPA was assessed via IR binding and cell-based assays to determine maximal IR signaling and metabolic responses relative to RHI and/or Lantus®. Insulin receptor binding assays were run using membranes prepared from CHO (ATCC; CCL-61) cell lines overexpressing hIR with [^125^I]-radiolabeled native insulin as the competitor ligand^50^.

Insulin stimulated phosphorylation of Akt (pAkt, HTRF assay kit) in cell-based assays was determined to be the most sensitive IR signaling assay for detecting partial agonism derived from the IRPA compounds. Partial agonism of IRPA was expressed as the percentage of maximal response relative to RHI in the CHO-hIR pAkt assay. Selective compounds were also tested in CHO (ATCC; CCL-61) cells expressing minipig (mpIR) or dog IR (dIR). The pAkt activity of IRPAs was also determined in primary mouse hepatocytes or differentiated human myotubes.

Potency and selectivity of pAkt and pERK (HTRF assay kit) signaling was tested in HEK293 (ATCC; CRL-1573) cells expressing IGF-1R under similar conditions. The mitogenicity potential of IRPA was measured by ^3^H-Thymidine uptake into SAOS-2/B10 cells following procedure described previously^36^.

Insulin receptor content (membrane IR density) of CHO-hIR cells was determined using Scatchard plot under saturation binding, with [^125^I]-radiolabeled native insulin as the competitor ligand (result not shown).

Insulin receptor internalization was quantified using DiscoverX PathHunter® U2OS INSR Internalization Assay (https://www.discoverx.com/products/cell-line/u2os-insrb-internalization-assay-93-0808c3) following manufacturer’s instructions.

### Cryo-EM studies

Experimental details can be found in^21^ and supplementary section of current manuscript.

### HDX-MS studies

The HDX-MS experiments were carried out using a soluble, fully glycosylated insulin receptor ectodomain b isoform produced in house. The protein solution (0.7 mg/mL, MW ~300 kDa) was concentrated to ~20 µM. To form IR:peptide complex, 10 µM IR was incubated with 100 µM insulin or IRPA at 4 °C overnight. HDX-MS experiments were performed using an automated HDX system (Waters Corporation, MA, USA). Six microliters of IR or IR:peptide stock solution were diluted 5-fold with labeling buffer at 20 °C to initiate the deuterium exchange reaction. The labeling time points were 0, 10 s, 30 s, 1 m, 15 m, 1 h and 4 h. At each time point, an aliquot was removed and an equal volume of quench buffer (3 M urea, 0.5 M TCEP in 100 mM phosphate buffer, pH 2.5) was mixed at 4 °C and immediately analyzed. Online digestion was performed using an immobilized pepsin column, 2.0 ×30 mm (Applied Biosystems, CA, USA). The eluent was directed into a SYNAPT® G2 HDMS mass spectrometer for analysis in MS(e) mode over the m/z range of 300-2000. Data acquired from undeuterated samples including four replicates of IR, IR:insulin and IR:IRPA were used to identify peptides through PLGS 3.0 software. Raw data from all time points were analyzed using DynamX 3.0 to generate relative deuterium uptake level in each peptide, which was used to generate deuterium uptake graphs and difference maps.

### Rodent studies

All animal procedures described in this paper were reviewed and approved by the research laboratories of Merck & Co., Inc., Kenilworth, NJ, USA, Institutional Animal Care and Use Committee. The Guide for the Care and Use of Laboratory Animals was followed in the conduct of the animal studies. All animals were housed in a room maintained at constant temperature (23 °C ± 2 °C), and humidity (55% ± 15%) under controlled conditions of 12-hour light/12-hour dark cycles with ad libitum access to autoclaved water and diet.

Male C57BL/6 mice and Sprague-Dawley rats were obtained from Taconic Farms (Germantown, NY, USA). AAV-LIRKO mice were developed by AAV-Cre injection into conditional IR KO mice (B6.129S4(FVB)-Insr^tm1Khn^/J from Jackson Laboratory; Fig. S3). To generate streptozotocin (STZ)-induced diabetic mouse, male C57BL/6 mice were treated with a single intraperitoneal (i.p.; 100 mg/kg) injection of STZ (Sigma Chemical, St. Louis, MO, USA).

### IRPA-mediated tissue signaling and metabolic efficacy

To test agent’s acute glucose lowering effects, mice were fasted for 2 h prior subcutaneous (s.c.) injection of IRPA or RHI. Glucose was measured in blood from tail bleeds using a OneTouch glucometer (Lifescan, Milpitas, CA; USA) at specified time points after insulin injection. Plasma was collected at 0, 30, 60, 120 or 180 min post dosing. RHI/IRPA levels were measured by ELISA according to manufacturer’s protocol (Mercodia, Salem, NC, USA). Concentration of insulin analogs were calculated based on standard curves derived from the specific compounds.

Healthy normoglycemic C57BL/6 mice were studied under the same conditions to compare insulin signaling using phospho-Akt (pAkt; Ser 473; Meso Scale Discovery, MD, USA). Insulin (Levemir or IRPAs) was dosed i.p. 4 h after food removal. 120 min after injection, liver and gastrocnemius muscle were collected and snap frozen in liquid nitrogen for phospho-protein analysis.

### IRPA interstitial distribution quantification

Male Sprague-Dawley rats were surgically prepared 24–48 h prior to dosing. The cannulation site was located at the thoracic lymph duct near the diaphragm inside the upper abdomen. The duct was cannulated with a heparin saline filled catheter. A modified catheter system was used which has two catheters combined with a mixing chamber. Micro infusion of heparin (500 units/mL) was applied from the second catheter into the mixing chamber at a constant flow rate of 50 μL/h. On average the lymph flow rate was at 2.2 ± 0.98 mL/h. The s.c. dose of IRPA or RHI was injected at the lateral lower part of the left hind limb of the animal.

### Glucose lowering in diabetic minipig

Minipig studies described in this paper were reviewed and approved by the Merck Research Laboratories, Kenilworth, NJ, USA, and Sinclair Research Center, Auxvasse, MO, USA, Institutional Animal Care and Use Committees. The Guide for the Care and Use of Laboratory Animals was followed in the conduct of the animal studies. All animals were housed in a room maintained at constant temperature (23 °C ± 2 °C), and humidity (55% ± 15%) under controlled conditions of 12-h light/12-h dark cycles with ad libitum access to water. Diabetic minipigs were fed twice a day and glucose levels maintained with twice daily doses of neutral protamine Hagedorn (NPH) insulin.

IRPAs were characterized in a Type 1 diabetic Yucatan minipig model. Briefly, male Yucatan minipigs, from Sinclair, were rendered Type 1 diabetic by Alloxan injections following a proprietary protocol developed by Sinclair Research Center (Auxvasse, MO). Induction was considered successful if basal glucose levels exceeded 150 mg/dL. Minipigs used in these studies ranged from 10 months to 3 years old, had plasma glucose levels of approximately 300–400 mg/dL and were instrumented with two Jugular vein vascular access ports (VAP). Glucose lowering was tested after intravenous (i.v.) and or s.c. administration of IRPA, recombinant human insulin (RHI; Humulin R® from Lilly) or Lantus® (insulin Glargine from Sanofi) administered i.v. or Tresiba® (insulin Degludec from Novo Nordisk) administered s.c.

#### Intravenous administration of IRPAs to type 1 diabetic minipigs

On the day of the study, after an overnight fast, minipigs were placed in slings, and VAPs were accessed for infusion and sampling. At *t* = 0 min, and after collecting two baseline blood samples for plasma glucose measurement (*t* = −30 min and *t* = 0 min), minipigs were administered RHI or IRPA as a single bolus i.v. RHI and IRPAs were administered at escalating (30% step-wise increase) doses. After dosing, sampling continued for 480 min. Blood was collected in K_3_-EDTA tubes, supplemented with 10 µg/ml aprotinin, and kept on ice until processing, which occurred within 30 min of collection. After centrifugation at 1000x*g*, 4˚C, for 8 min, plasma was collected and aliquoted for glucose measurement using a Beckman Coulter AU480 Chemistry analyzer.

#### Subcutaneous administration of IRPAs to type 1 diabetic minipigs

IRPAs were administered to diabetic minipigs by subcutaneous injection. Tresiba® (Insulin Degludec) was used as a comparator, at a dose selected to match potency. After dosing, sampling continued for 18 h.

### Euglycemic clamp in healthy beagle dogs

Dog studies described in this paper were reviewed and approved by the Merck Research Laboratories, Kenilworth, NJ, USA, Institutional Animal Care and Use Committee. The Guide for the Care and Use of Laboratory Animals was followed in the conduct of the animal studies. All animals were housed in a room maintained at constant temperature (23 °C ± 2 °C), and humidity (55% ± 15%) under controlled conditions of 12-h light/12-h dark cycles with ad libitum access to water. Dogs were fed once a day.

The healthy lean dog is a preclinical model that has been widely used to interrogate insulin pharmacology. Its translatability to humans is well documented. Male beagle dogs, 2–8 years old, from Marshall Bioresources, were used in these studies. A dog clamp paradigm used to assess and measure in vivo partial agonism as an overall systemic effect and on the basis of selective tissue-specific actions in liver, adipose and muscle (Fig. S6A). Euglycemic clamps with IRPA were conducted in healthy male beagles to examine its PD (GIR; GRI reference) across a range of doses. Dogs were fasted overnight prior to a clamp and had previously been prepared with dual-ported (femoral artery and femoral vein) cannulation.

To demonstrate in vivo partial agonism and potential TI improvement, euglycemic dog clamp studies of IRPA-1/7 and RHI were conducted under multiple doses of each, as shown in Fig. 4a and Fig. S5A, B. Steady state glucose infusion rate (GIR) that were required to sustain stable plasma glucose at the level of euglycemia at a given dose represents the corresponding pharmacodynamics.

Inclusion of glucose tracer (primed-infusion of [U-^13^C_6_]glucose, administered as an intravenous bolus at 6 mg/kg bolus followed by a constant infusion of 0.06 mg/kg/min) in the clamp study allows determination of liver vs. peripheral action in glucose metabolism as HGP and peripheral glucose disposal (Rd) respectively to minimize wide shifts in plasma glucose labeling, during the clamp we maintained the infusion of the baseline tracer and enriched the glucose infusate to 1.5%.

The ^13^C enrichment of glucose was determined using GC-MS as follows. Plasma samples (10 μL) were vigorously mixed after the addition of 100 μL of methanol. Following a brief centrifugation, the supernatant was removed and evaporated to dryness under a stream of nitrogen. The residue was reacted with 50 μL of hydroxylamine hydrochloride (25 mg/mL pyridine) at 65 °C for 30 min, and 50 μL of acetic anhydride was then added and the heating continued for another 30 min. Samples were then evaporated to dryness under a stream of nitrogen and the residue was dissolved in 70 μL of ethyl acetate before GC-MS analyses. Samples were analyzed in an Agilent 5973 mass spectrometer coupled with a 6890 N GC (HP 5 column, 175 °C initial temperature, hold for 0 min, and ramp to 300 °C at 35 °C per minute, hold for 1 min). Data were acquired using selected ion monitoring under electron impact ionization (m/z 314 to 319, 10 ms dwell per ion). Note that this analytical scheme uses an M + 5 fragment ion as a surrogate of the M + 6 tracer being infused; since we have not observed “recycling” of the primary tracer (i.e. no formation of M + 1, M + 2, etc.) in these studies the labeling of M + 5 reflects that of the infused glucose tracer.

### Formulation and PK studies

In the single and multiple dose PK studies, IRPA was formulated in F1 vehicle (16.0 mg/mL of glycerin, 1.6 mg/mL of m-cresol, 0.65 mg/mL of phenol, 26.5 mM phosphate at pH 7.4) containing 0.45 eq Zn^2+^ for both i.v. and s.c. administration. Plasma concentration of IRPA was measured by LC-MS/MS following immunoaffinity purification using a mouse anti-insulin monoclonal antibody.

IRPA was formulated in F1 vehicle without m-cresol or phenol for rodent studies. Plasma concentration of IRPA was measured using insulin ELISA (see above) with specific IRPA molecule as standard.

The pharmacokinetics of IRPAs were evaluated in Wistar Han rats, Beagle dogs and Yucatan minipigs following i.v. and subcutaneous s.c. dosing. Following s.c. administration, bioavailability was moderate in rats, dogs and minipigs (33-60%).

### Statistical analysis

Data analyses were performed in GraphPad Prism® (GraphPad Software, San Diego, CA). Calculations of p-values were based on analysis of variance (ANOVA) or the unpaired student’s *t* test when applicable, unless otherwise specified. Statistical significance was defined as *p* < 0.05.

### Reporting summary

Further information on research design is available in the Nature Research Reporting Summary linked to this article.

### Supplementary information

Supplementary Information

Reporting Summary

### Source data

Source Data

## Data Availability

Source data are provided with this paper in Source Data Excel file or a hyperlink to public storage. The cryo-EM maps of insulin receptor complexed with IRPAs are deposited to the Protein Data Bank (https://www.rcsb.org/) under accession codes 7MD4 and 7MD5, respectively. All the deuterium uptake plots of the experiments presented for insulin receptor are available on figshare data repository using the following link: (10.6084/m9.figshare.17102741). Source data are provided with this paper.
