# Author Correction: Functionally selective signaling and broad metabolic benefits by novel insulin receptor partial agonists

**DOI:** 10.1038/s41467-024-44938-4

**Published:** 2024-01-23

**Authors:** Margaret Wu, Ester Carballo-Jane, Haihong Zhou, Peter Zafian, Ge Dai, Mindy Liu, Julie Lao, Terri Kelly, Dan Shao, Judith Gorski, Dmitri Pissarnitski, Ahmet Kekec, Ying Chen, Stephen F. Previs, Giovanna Scapin, Yacob Gomez-Llorente, Scott A. Hollingsworth, Lin Yan, Danqing Feng, Pei Huo, Geoffrey Walford, Mark D. Erion, David E. Kelley, Songnian Lin, James Mu

**Affiliations:** 1 Merck & Co., Inc., Kenilworth, NJ 07033 USA; 2 Merck & Co., Inc., South San Francisco, CA 94080 USA; 3 Merck & Co., Inc., Boston, MA 02115 USA

**Keywords:** Type 2 diabetes, Receptor pharmacology

Correction to: *Nature Communications* 10.1038/s41467-022-28561-9, published online 17 February 2022

This Article contains an error in the spelling of the author Yacob Gomez-Llorente, which is incorrectly given without hyphenation as Yacob Gomez Llorente. The error has been corrected in the PDF and HTML versions of the Article.

