# Supplementary material for: Functionally selective signaling and broad metabolic benefits by novel insulin receptor partial agonists
Source: Nat Commun. 2022 Feb 17;13:942. doi: 10.1038/s41467-022-28561-9 (PMC8854621; doi:10.1038/s41467-022-28561-9)
Supplement: Supplementary file 1 — Supplementary Information [file 41467_2022_28561_MOESM1_ESM.pdf]

Functionally Selective Signaling and Broad Metabolic Benefits by Novel Insulin Receptor Partial Agonists

[1*]Margaret Wu, [1*]Ester Carballo-Jane, [1*]Haihong Zhou, [1]Peter Zafian, [1]Ge Dai, [2]Mindy Liu, [2]Julie Lao, [1]Terri Kelly, [2]Dan Shao, [1]Judith Gorski, [1]Dmitri Pissarnitski, [1]Ahmet Kekec, [1]Ying Chen, [1]Stephen F. Previs, [1]Giovanna Scapin, [1]Yacob Gomez Llorente, [2]Scott A. Hollingsworth, [1]Lin Yan, [1]Danqing Feng, [1]Pei Huo, [3]Geoffrey Walford, [1]Mark D. Erion, [1]David E. Kelley, [1]Songnian Lin, and [2]James Mu

[1]Merck & Co., Inc., Kenilworth, NJ, 07033, USA.

[2]Merck & Co., Inc., South San Francisco, CA 94080, USA.

[3]Merck & Co., Inc., Boston, MA 02115, USA

*Equal contribution

Address correspondence to: James Mu, 213 East Grand Ave., South San Francisco, CA 94080, USA. Email: yingjun_mu@merck.com; Phone: 650.496.4507; Songnian Lin, 2015 Galloping Hill Road, Kenilworth, NJ 07733, USA. Email: Songnian.lin@merck.com; Phone: 908.740.0585.

Supplementary Material and Methods

1. Synthesis of IRPA

RHI was used for the synthesis of dimers, except for IRPA-4, for which desB30 B3K B29R RHI was used. Briefly, IRPA-1 was synthesized in a two-step process, where the first step included bis-carbamylation of A1 and B1 sites of insulin with potassium isocyanate in water. The second step included dimerization using disuccinimidyl suberate in organic solvent with triethylamine as the base. Similar chemistry was used for the synthesis of IRPA-2, IRPA-3, IRPA-7, IRPA-8. Compounds with triazole (Trz) linker, IRPA-4, IRPA-5, IRPA-6, and IRPA-9 were assembled by copper (I) catalyzed "click" reaction from individually prepared alkyne- and azide-decorated insulins. The synthesis of IRPA-1 and IRPA-4 are described below in detail. The experimental details for the preparation of the rest of the IRPA dimers are similar to those described for IRPA-1 and IRPA-4 and can be found in literature [1,2] as well as medicinal chemistry publications being prepared.

Synthesis of IRPA-1 (MK-5160).

Step 1. Synthesis of N2,1A,N2,1B-bis(carbamoyl) RHI.

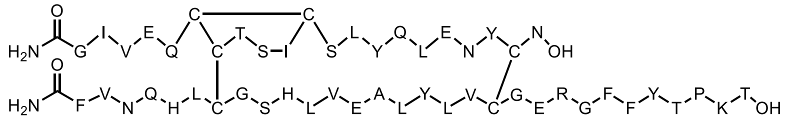

To a suspension of RHI (1g, 0.172 mmol) in water (50 mL) was added a solution of potassium phosphate, dibasic (0.249 g, 1.429 mmol) in water (5.0 mL). After stirring at room temperature for 30 minutes, to the resulting mixture was added potassium cyanate (0.279 g, 3.44 mmol). The reaction mixture was allowed to stir for 16 hours. To stop the reaction, unreacted potassium cyanate was removed by TFF using MWCO 3K diafiltration device, and the product was isolated as a solid by lyophilization. The product contained about 10-35% of A1/B1/B29-tris-urea-

RHI, which optionally could be removed by preparative HPLC with gradient 26-34% (Solvent A: Water-0.05%TFA; Solvent B: AcN-0.05%TFA). UPLC-MS Method C: Rt = 4.29 min, m/z = 1474.6 (z = 4). Average yields of desired material ranged from 30 to 70%.

Step 2. Synthesis of IRPA-1 by dimerization with bis-functional linker.

Dissolved the product of previous step (1.488 g, 0.252 mmol) in DMSO (21 mL) and added 1.06 mL (7.57 mmol) of triethylamine followed by dropwise solution of disuccinimidyl suberate (0.037 g, 0.101 mmol) in 1.0 mL of DMSO. The mixture was stirred for 30 min and dimerization was verified by UPLC. The reaction mixture was added dropwise to 20 volumes of water and pH adjusted to 7.4 by addition of 1M HCl. Concentrated the reaction mixture by diafiltration to volume of ~50 mL. The product was isolated by reverse-phase chromatography using 26-40% gradient (Solvent A: Water-0.05%TFA; Solvent B: AcN-0.05%TFA) in 30 min, 10 sequential portion-wise injections were done for the purification of the total batch. 550 mg (36%) was obtained after lyophilization of fractions. UPLC-MS Method D: Rt = 3.59 min, m/z = 1988.4.6 (z = 6).

Synthesis of IRPA-4.

Step 1. Synthesis of the B3 alkyne component.

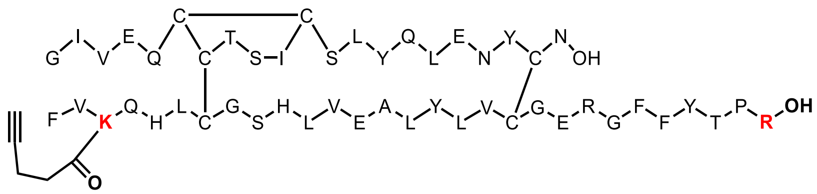

Dissolved desB30 B3K B29R RHI 200 mg (0.035 mmol) in a mixture containing 1.8 mL of 0.1M Na_2CO_3 and 1.2 mL of AcN and adjusted pH to 10.6 using 1N NaOH. Added 1-(pent-4-ynoyl)pyrrolidine-2,5-dione as a solid and stirred the reaction mixture for 3 h. Diluted the reaction mixture with 7 mL of water, adjusted pH to 3.55. The product was isolated by preparative HPLC,

gradient 27-35% in 30 min (Solvent A: Water-0.05%TFA; Solvent B: AcN-0.05%TFA). Obtained 100 mg (49%), UPLC-MS Method C: Rt = 4.45 min, m/z = 1458.1 (z = 4).

Step 2. Synthesis of the B3 azide component.

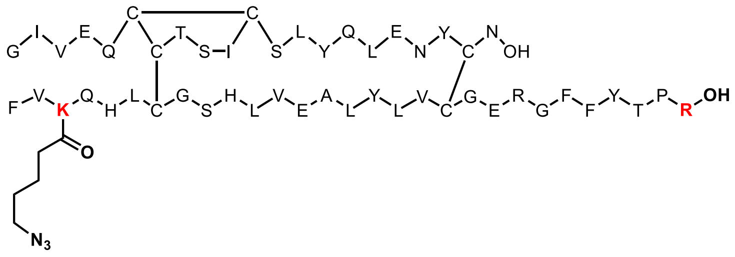

This step was performed analogously to Step 1, except that 1-(5-azidopentanoyl)pyrrolidine-2,5-dione was used as the acylating agent. UPLC-MS Method C: Rt = 4.62 min, m/z = 1469.4 (z = 4).

Step 3. Synthesis of IRPA-4 by Dimerization via “click” reaction.

A mixture containing 50 mg (8.6 μmol) of alkyne component, 50 mg (8.6 μmol) of azide component, 12 mL of DMSO, 18 mL of water, and 7.0 mL of 2M of triethylammonium acetate buffer, PH=7.0, was degassed by nitrogen purge for 10 min. Added 4.0 mL of freshly prepared 5.0 mM aq. ascorbic acid solution and degassed by nitrogen purge for 1 min. Added 2.0 mL of 10 mM $CuSO_4$-TBTA (55% DMSO-water) solution and degassed by nitrogen purge for 1 min. Gently shook the mixture overnight. The precipitated product was re-dissolved by addition of 80 mL of 30% AcN-water solution and adjustment pH to 3.5. The mixture was concentrated to total volume of 8.0 mL in Amicon tubes and the product was isolated by preparative HPLC using 25-45% gradient (Solvent A: Water-0.05%TFA; Solvent B: AcN-0.05%TFA) in 30 min. After lyophilization of fractions 9.0 mg (9%) of product was obtained. UPLC-MS Method D: Rt = 4.27 min, m/z = 1951.8 (z = 6).

2. **Cellular assays to profile site-specific phosphorylation**

CHO-hIR cells were serum starved overnight, then treated with compounds at 37 ^{o}C for 10 minutes. The cells were lysed with lysis buffer (MSD) on ice in the presence of protease inhibitors

and phosphatase inhibitors for 30 minutes. The lysates were applied to the modified MSD Elisa assays to measure phosphorylation of different tyrosine residues on insulin receptor. Briefly, lysates were added to custom MSD IR plates (MSD cat# N45CA-1). Phosphorylation of tyrosine on IR were measured using antibody against pY960 (rabbit mAb, Cell Applications #CB4378) or pY1345 (rabbit mAb 14A4, Cell Signaling #3026), respectively in combination with SULFO-tag anti rabbit antibody (MSD cat# R32AB). For pY1150, cell lysate was added to MSD high binding plate (MSD cat # L15XB) coated with pY1150/1151 antibody (mouse monoclonal antibody 19H7, Cell Signaling #3024). The bound insulin receptor was detected using combination of rabbit anti-insulin receptor b (C-19, Santa Cruz sc-711) and SULFO-tag anti rabbit antibody.

3. **IGF-1R binding, cellular proliferation in SaoS2 cells and 2-deoxyglucose (2DG) update in mouse adipocytes**

IGF-1R affinity of IRPAs was assessed in a whole cell binding assay using a cell line stably expressing human IGF-1R with radiolabeled native IGF-1 as the competitor ligand. Cellular proliferation activities of IRPAs as measured by EdU incorporation to SAOS2/B10 cells using the Click-it EdU (5-Ethynyl-2′-deoxyuridine; ThermoFisher Scientific) assay. To assess IRPA function downstream of the signaling cascade, [^{14}C]-2-deoxyglucose cellular uptake in the differentiated mouse 3T3 L1 adipocytes was measured using standard protocol ([3]).

4. **Cryo-EM studies**

Electron microscopy

The preparation, collection and processing of the sample of IR in complex with RHI was performed as described in [4]. The complexes with IRPA-3 and IRPA-9 were made by mixing the purified IR

ectodomain at a concentration of 0.3 mg/mL with a 10 molar excess of the different synthetic insulin molecules and incubating at 4°C for 1 h. For every sample, 3 μL were applied to grids previously plasma cleaned with a Solarus (Gatan). To prevent the preferred orientation problem typically observed with this protein, a combination of different grids were frozen by using different available techniques: both for IRPA-3 and IRPA-9, nanowire-Lacey grids were ultra-fast frozen with SpotItOn (at the Simons Electron Microscopy Center in the New York Structural Biology Center); additionally for IRPA-3, grids were plunge-frozen using both 1.2/1.3 C/Cu C-flat or UltraAu Au/Au and a manual cryoplunger (at NanoImaging Services). The C/Cu and SpotItOn grids were used to collect untilted data and the Au/Au grids to collect -30 deg tilted data at NIS in a 300 kV Titan Krios (ThermoFisher) equipped with a GIF Quantum 967 LS imaging filter (Gatan) and a K2 Summit direct detector (Gatan). Movies were recorded in counting mode at a calibrated pixel size of 1.04 Å/pix. The movies were fractionated into 30 frames of 200 ms (yielding movies of 6 s duration) and a total accumulated exposure of 45 $e^{-}/Å^{2}$. For IRPA-3 a total of 1673 movies were collected without tilt for C/Cu grids, 5778 movies from SpotItOn grids and finally 5633 movies for Au/Au applying -30 deg tilt. In each case, they were necessary several sessions to collect these amounts. For IRPA-9, a total of 2974 images were collected in a single session.

Image processing and structure determination

Images for all datasets were collected using Leginon. The frame alignment was carried out using MotionCor2 and their CTF was calculated per-particle using gCTF. After a strict data evaluation based on their CTF information and visual inspection 46%, 38% and 66% of the movies were discarded for IRPA-3 grids of C/Cu, SpotItOn and Au/Au respectively, while 5% of the movies were discarded for IRPA-9 grids. For IRPA-9, particle picking done using Gautomatch (https://www2.mrc-lmb.cam.ac.uk/research/locally-developed-software/zhang-software/) resulting in 1,236k particles that were extracted by using Relion. The extracted particles were

imported and processed (from 2D classification to final reconstruction) in cryoSPARC v0.6.5. After 2 rounds of 2D classification, 212k particles were selected for an ab-initio reconstruction with 5 classes and without imposing symmetry, in which one of the resulting volumes accumulated 47% of the particles and showed the best quality and features of the expected C2 symmetry. The corresponding volume was refined thorough a single homogenous refinement job and using the associated particles to a 5.2 Å resolution map.

For IRPA-3, all the remaining movies after CTF evaluation were reimported into cryoSPARC v2, realigning their frames and re-estimating their CTF with GCTF. A resulting dataset of 692k particles was selected for local motion correction and after 3 rounds of 2D classification, reduced to 258k particles that were fed to an ab-initio job for 3 classes without imposing symmetry. The 2 best classes were selected based on their 3D features and angular coverage and their particles used to start an iterative series of several ab-initio jobs with the same parameters than the initial one. For every step of that series, the particles were used both to run a subsequent ab-initio job and a homogenous refinement, and the refined volume inspected in terms of FSC resolution and 3D features. At the end of the 5th round, 92k particles until a final dataset of 92k particles resulted in the best reconstruction at 5.0 Å of resolution. The resulting volume and particles were refined by C2 non-uniform refinement generating the final map at 4.5 Å of resolution, calculated by gold-standard FSC.

The previously solved cryoEM complex of IR bound to insulin was positioned into both maps using MolRep and rigid-body refinement using Coot. The final structures were subjected to several cycles of global real-space refinement in Phenix with the parameters NCS, rotamer, Ramachandran plot and C-beta deviations restraints enabled. There was no density in the map of

IRPA-9 for the C-terminal residues 590 to 806 of one of the chains, being that part of the polypeptide chain modeled based on similarity with the insulin bound complex structure. Map visualization was done by using Chimera and figures both by using Chimera and Pymol (The PyMOL Molecular Graphics System v.1.8). Data collection, refinement and validation statistics are summarized in Table S3.

5. **HDX-MS Studies**

The HDX-MS experiments were carried out using a soluble, fully glycosylated IR-ectodomain b isoform. IR-ectodomain an isoform was also tested (R&D Systems Cat# 1544-IR/CF). Both isoforms yielded similar H/D signatures upon insulin binding (data not shown), demonstrating that both isoforms have similar conformation in solution. Thus, for structural studies, both isoforms can be used to understand the interaction behaviors of human insulin and its analogues. Typical sequence coverage for IR(b) or IR(a) is approximately 77% with greater than 250 peptic peptides detected (Fig. S2). Reasonable sequence coverage was obtained for almost all their-ECD domains except cysteine-rich domain.

Difference in deuterium uptake rate was detected when comparing IR-ECD alone to IR-ECD bound with RHI, IRPA-3 or IRPA-9. The sequence regions that showed deuterium uptake difference for RHI or IRPA-9 bound IR-ECD are listed in Table S1, and for IRPA-3 bound in Table S2. A H/D difference plot is generated by the sum of difference in deuterium uptake rate for each peptic peptide at each deuterium labeling time point between bound and unbound IR-ECD (y-axis) and peptic peptides are arranged from N to C terminus along X-axis (Fig. 2i). The grey bar pointing downward indicates that the deuterium uptake rate is reduced upon ligand binding. The magnitude of the grey bar (y-axis) greater than (± 0.5 Da) is considered statistically significant.

6. **Mouse models and related studies**

LIRKO mouse model generation

IR^{lox} mutant mice on C57BL/6 background have loxP sites flanking exon 4 of the INSR gene (The Jackson Laboratory, #6955). Male mice at 8 weeks of age maintained on chow diet (Teklad 7002) were administered AAV-cre via tail vein injection as doses indicated on Fig. S4. Mice were studied 4 weeks following AAV injection for ITT or put on HFD (Research Diets, D12492) for 4 weeks before glucose lowering studies were executed.

IRPA studies in HFD-STZ mice

HFD-STZ mice were developed as previously described [40] and were groups based on their glucose ranges. Acute glucose lowering efficacy of IRPAs in healthy and HFD-STZ mice were evaluated upon administration of escalating doses of test molecules, which was expressed as area over the curve (AOC) relative to vehicle.

7. **Dog clamp platform**

Intact, male Beagle dogs (2-8 years old) were either purchased from Marshall Bioresources or acquired from other in-house programs. Beagle dogs were trained to stand in canvas slings (modified with Velcro® straps) mounted on stainless steel frames that were modified with a standing platform (LOMIR Biomedical Inc., Canada). Vascular access ports (VAP; venous and arterial, Access Technologies; CP2AC or Swirl-MaxAC-7NC with 7 French Intisil, round-tip catheter; dead space approx. 1.7 ml) were surgically implanted in the right femoral artery and vein minimally 4-6 weeks before studies. For maintenance purposes, VAPs were flushed every two weeks with a 20U/mL heparinized saline solution and locked using TCS (Taurolidine-Citrate; Access Technologies). If the VAPs implanted in the right femoral artery and vein became clogged

or difficult to draw blood from, VAPs would then be implanted in the left femoral artery & vein and utilized instead.

Beagle dogs were fasted overnight (18-20 hrs) prior to the clamp procedure. All Beagle dogs were prescreened with Humulin-R base clamps (3pmol/kg/min; all procedures similar to unknown insulin clamps explained below) to assess relative insulin sensitivities and arranged in response-balanced cohorts of four. The morning of the clamp, Beagle dogs were placed in slings. Using aseptic techniques, a Huber-needle extension line (Access Technologies) was inserted into each VAP for blood draws and tracer-enriched dextrose infusion (arterial and venous VAPs, respectively). Blood was drawn from subject's arterial VAP and each of their own isolated plasma was used to prepare individualized protein-hormone infusates using sterile saline (2% plasma in solution). A pre-clamp hematocrit was measured at this point. A temporary infusion catheter was also placed into the cephalic vein (alternated left and right between studies) for all other infusates. A bolus of 6 mg/kg of [U-$^{13}C_6$]glucose (T= -90min, 13C6D in saline; Sigma Aldrich #389374, or Cambridge Isotope Laboratories, Inc. #CLM-1396; both 99 atom %) was given into the cephalic vein followed by a constant infusion of 0.06mg/kg/min of ^{13}C6D for the remainder of the clamp procedure (~ 6hrs). The pre-clamp tracer infusion continued for 90 min and blood was drawn for 3 successive baseline evaluations of tracer, plasma glucose, glucagon and C-peptide during this time (T= -20, -10, 0). All clamp solutions & infusates' concentrations were prepared & adjusted to each patient's body weight (BW, kg) to avoid introducing significant systemic effects over time [5] and because of differences across cohort BWs. After baseline samples were taken, Beagle dogs are first infused with a ramped somatostatin infusion (Bachem H-1490, 2 min prior to any other solution;) at 1000 μL/min for 2 min then set at a final rate (based upon BW) for the remainder of the experiment (0.8 μg/kg/min). A glucagon infusion (0.8 ng/kg/min) is also started to add basal tone to the liver (Lilly Glucagon Emergency Kit; NDC 0002-8031-01, MS8031; 1mg per

vial). Experimental insulin infusates were prepared and infusion started (T=0) as a ramped bolus infusion (~16-18% expected total insulin load) over the first 10 min and then set at 200 μL/min for the remainder of the experiment. ^{13}C6D-enriched Dextrose (1.5% enrichment in Hospira 45-46% dextrose) is used to clamp plasma glucose at 100 mg/dL (GM9 glucose analyzer, ANALOX Instruments) and to track endogenous glucose production.

Beagle dogs were clamped for 3 hours. Plasma glucose was measured every 15 min. Larger plasma samples (aprotinin treated) were also collected at +60, 120, 135, 150, 165, 180, 210 & 240 min for glucagon and C-peptide measurements while neat plasma was used for endogenous glucose production estimations. At T=+180 unknown insulin infusions were halted and Beagle dogs were clamped for another 60 min. At T=+240 glucagon & somatostatin infusions were halted and a final hematocrit taken. Beagle dogs were fed and supported with infused dextrose until stabilized. Beagle dogs were rested for minimally 4 weeks before reuse. Unknown insulins were initially tested at infusion rates of 6 & 9 pmols/kg/min (pkm); expanded to 3, 6, 9 12, 15-16 and 20 pkm as necessary. C-peptide and glucagon measurements were made using Millipore's C-peptide & Glucagon RIA kits (CCP-24HK & GL-32K, respectively). Glucagon assays were altered by adding an additional 24hrs incubation prior to the delayed addition of labeled tracer.

Supplementary Table

Table S1. Comparison between HDX Signatures and Cryo-EM Map for Insulin Receptor Ectodomain Complexed with RHI or IRPA-9.

IR L1	IR α-CT helix	IR L2	IR FnIII-1	IR FnIII-2
HLQILLMF (32-39)	CSCPKTDSQILKE (683-695)+	IDSVTSAQELRG (321-332)	SYIRTSF (476-482)	LKGLKL (648-653)+
LMFKTRPED (37-45)	LEESSF (696-701)+	IRGGNN (344-349)	EPYWPPDF (490-497)	DPRPS (724-731)
ITDYLLL (57-63)*	FRKTFEDYLHN (701-711)	RSYAL (372-376)	RDLLGFM (498-504)*	SVAA (808-811)+
LFRVYGL (63-69)*	HNVVF (710-714)		PLRSNDPKSQNHPGWL (537-552)	
VIRGSRL (81-87)			KTLVTFSDE (567-575)+	
FFNYALVIFEM (88-96)				
V99				
RIEKNNEL (118-125)*				
R14, R188			R498	

Underlined letter: interaction site observed in the cryoEM map. Asterisk: peptic peptide containing cryoEM interaction site but shows no H/D difference (low H/D exchange occurred under the current experimental conditions, preventing the detection of any difference in deuterium uptake); Plus sign: increased H/D exchange upon insulin binding; Red: residues observed in cryoEM map but no sequence coverage in HDX-MS studies. Blue: Regions not observed in IRPA-3:IR-ECD binding.

Table S2. Comparison between HDX Signatures and Cryo-EM Map for Insulin Receptor Ectodomain Complexed with IRPA-3.

IR L1	IR α-CT helix	IR L2	IR FnIII-1	IR FnIII-2
HLQILLMF (32-39)	CSCPKTDSQILKE (683-695)$^+$		TNGDQASCE (461-469)	LKGLKL (648-653)$^+$
LMFKTRPED (37-45)	LEESSF (696-701)$^+$		SYIRTSF (476-482)	DPRPS (724-731)
ITDYLLL (57-63)*	FRKTFEDYLHN (701-711)		FMLFYKEA (503-510)	
LFRVYGL (63-69)*	HNVVF (710-714)		PLRSNDPKSQNH PGWL (537-552)	
V99				
RIEKNNEL (118-125)$^+$				
R14, R188			DKILLR (483-488); MRG (553-555)	

Underlined letter: interaction site observed in the cryoEM map; Asterisk: peptic peptide containing cryoEM interaction site but shows no H/D difference (low H/D exchange occurred under the current experimental conditions, preventing the detection of any difference in deuterium uptake); Plus sign: increased H/D exchange upon insulin binding; Red: residues observed in cryoEM map but no sequence coverage in HDX-MS studies.

Table S3. Cryo-EM data collection, refinement, and validation statistics

	IRPA-3 partial agonist structure<br>(EMDB-23766)<br>(PDB 7MD4)	IRPA-9 full agonist structure<br>(EMDB-23767)<br>(PDB 7MD5)
**Data collection and processing**		
Magnification	130,000	130,000

Voltage (kV)	300	300
Electron exposure (e–/Å^2)	45.14	45.49
Defocus range (μm)	-1.0 to -2.5	-1.0 to -2.5
Pixel size (Å)	1.04	1.04
Symmetry imposed	C2	C2
Initial particle images (no.)	598,705	1,236,127
Final particle images (no.)	92,327	54,293
Map resolution (Å)	4.5	5.2
FSC threshold	0.143	0.143
**Refinement**		
Initial model used (PDB code)	4ZXB	4ZXB
Model resolution (Å)	3.3	3.3
R.m.s. deviations		
Bond lengths (Å)	0.01	0.01
Bond angles (°)	1.17	0.97
C-beta deviations (%)	0.0	0.0
CaBLAM outliers (%)	3.8	6.7
Validation		
Clashscore	16.4	8.3
Rotamer outliers (%)	0.06	0.00
Ramachandran plot		
Favored (%)	88.4	88.1
Allowed (%)	11.4	11.7
Disallowed (%)	0.24	0.22

References

1 Deppe, C., Breiner, M., Brandenburg, D. & Joost, H. G. Structure-Activity Relationship of Covalently Dimerized Insulin Derivatives - Correlation of Partial Agonist Efficacy with Cross-Linkage at Lysine B29. *N-S Arch Pharmacol* **350**, 213-217 (1994).

2 Lin, S., Yan, L., Huo, P., Pissarnitski, D., Feng, D., Nargund, R., Zhu, Y., Kekec, A., Madsen-Duggan, C. S., Shi, Z., Wu, Z., Mu, Y. Insulin receptor partial agonist. . WO2016081670 (2016).

3 Zhang, B. *et al.* Discovery of a small molecule insulin mimetic with antidiabetic activity in mice. *Science* **284**, 974-977, doi:10.1126/science.284.5416.974 (1999).

4 Scapin, G. *et al.* Structure of the insulin receptor-insulin complex by single-particle cryo-EM analysis. *Nature* **556**, 122-125, doi:10.1038/nature26153 (2018).

5 Mann, W. A. & Kinter, L. B. Characterization of maximal intravenous dose volumes in the dog (Canis familiaris). *Gen Pharmacol* **24**, 357-366, doi:10.1016/0306-3623(93)90317-q (1993).

Supplementary Figures

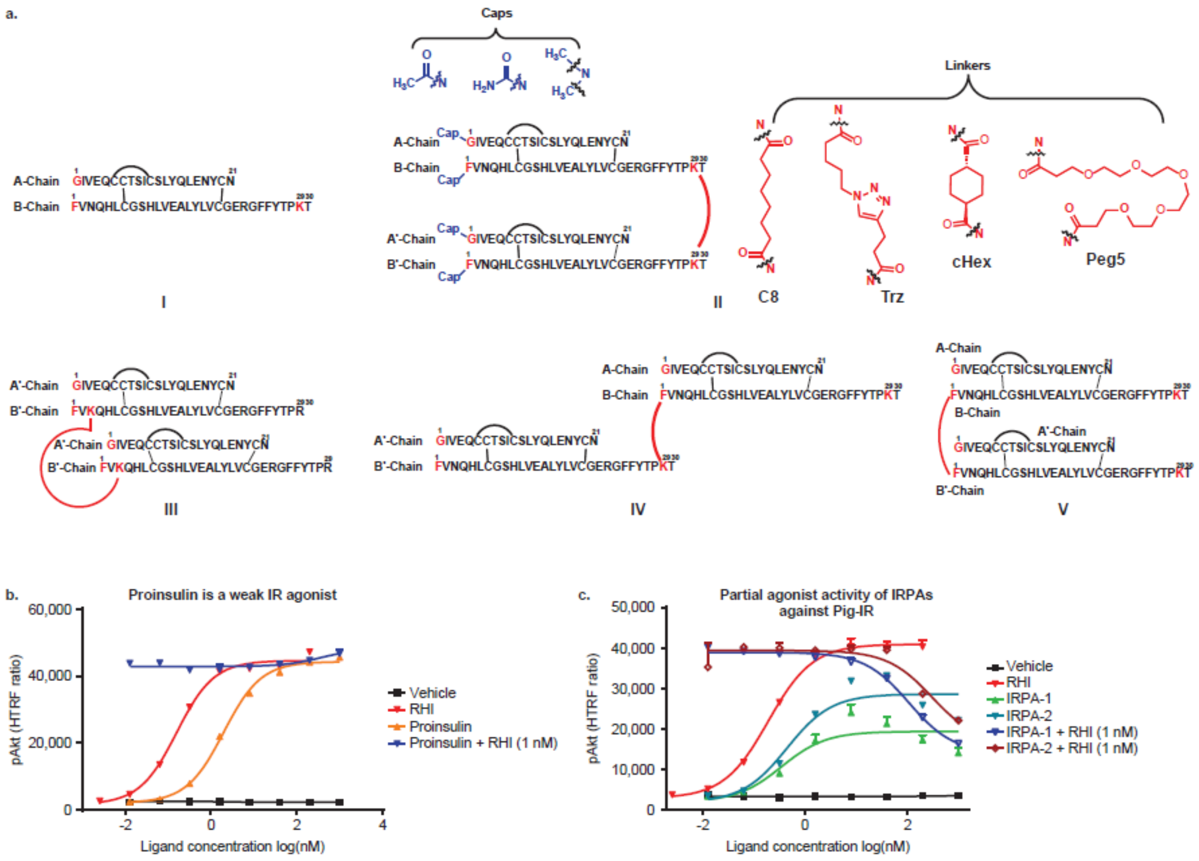

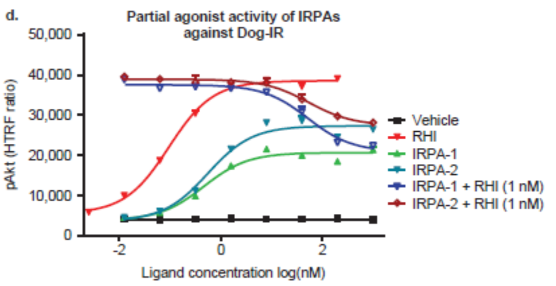

e.

IR site-specific phosphorylation by IRPAs in CHO-IR cells

		RHI (100%)	IRPA-3 (% vs RHI)	IRPA-6 (% vs RHI)
Juxtamembrane region	pY960	100	3	27
Kinase region	pY1150	100	9	36
Carboxyl-terminus	pY1345	100	1	26

Figure S1

Figure S1. Selected Covalent Insulin Dimers Function as Partial Agonists and Induce Biased Signaling. Schematic structures of RHI and IRPA dimers (S1a). I. Structure of native insulin. II. Structures of B29-B29' dimers, linkers and capping groups used. III. Structure of B3-B3' dimer. IV. Structure of B1-B29' dimer. V. Structure of B1-B1' dimer. Effect of proinsulin on pAkt in CHO-hIRb cells in agonist and antagonist mode (fixed RHI dose; S1b). IRPA-1 and IRPA-2 function as partial agonists and competitive antagonists against pig (S1c) and dog (S1d) IR in CHO cells expressing corresponding species-specific insulin receptor. All titrations in b-d were conducted in duplicate (mean ± SD). Emax of site-specific IR phosphorylation induced by IRPA (S1e) measured by MSD assays, developed using pY960 (Cell Application #CB4378), pY1150 (Cell Signaling #3024 (19H7)) or pY1345 (Cell Signaling #3026 (14A4)) specific antibodies. Source data are provided as a Source Data file.

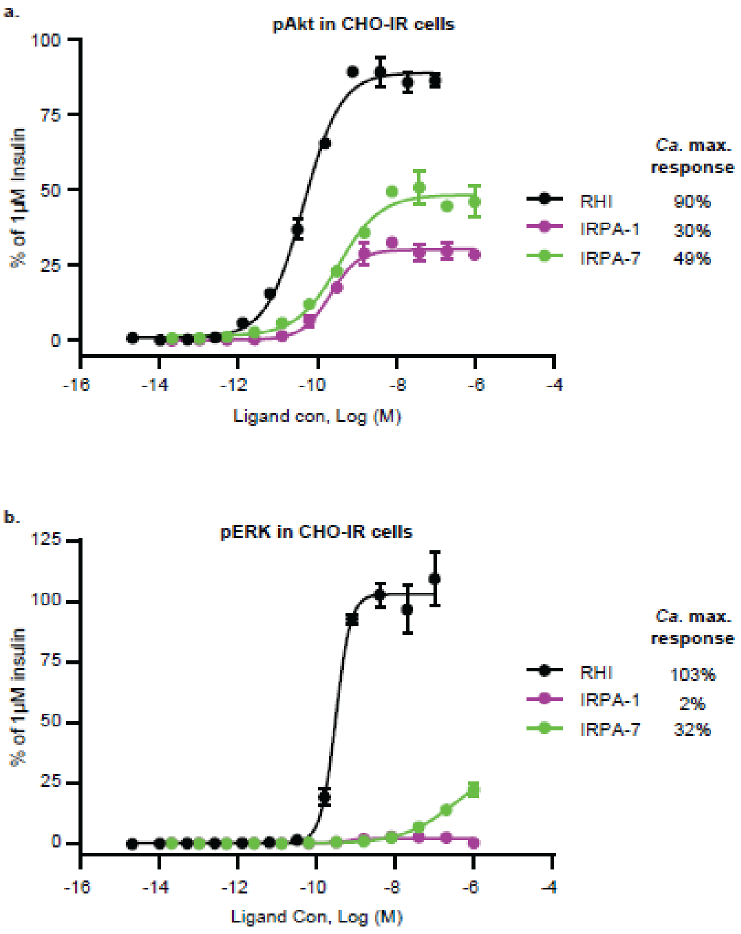

c.

IGF-1R binding and cellular proliferation

Cmpd	IGF-1R binding (EC_{50}; nM)	Cell proliferation (SaoS2 cells) (EC_{50}; nM)
RHI	134	6.5
IGF-1	0.46	0.15
IRPA-1	33,500	3940
IRPA-7	60,700	1910

Figure S2

Figure S2. IRPA signaling bias in CHO-IR cells, IGF-1R binding and mitogenesis activity. Potency and selectivity of pAkt (S2a) and pERK (S2b) signaling was tested in CHO cells expressing hIR using respective HTRF analysis. All titrations were conducted in duplicate (mean ± SD). IGF-1R binding affinity and cell proliferation (in SaoS2/B10 cells) activity of RHI and representative IRPAs were measured as described in Methods (S2c). Source data are provided as a Source Data file.

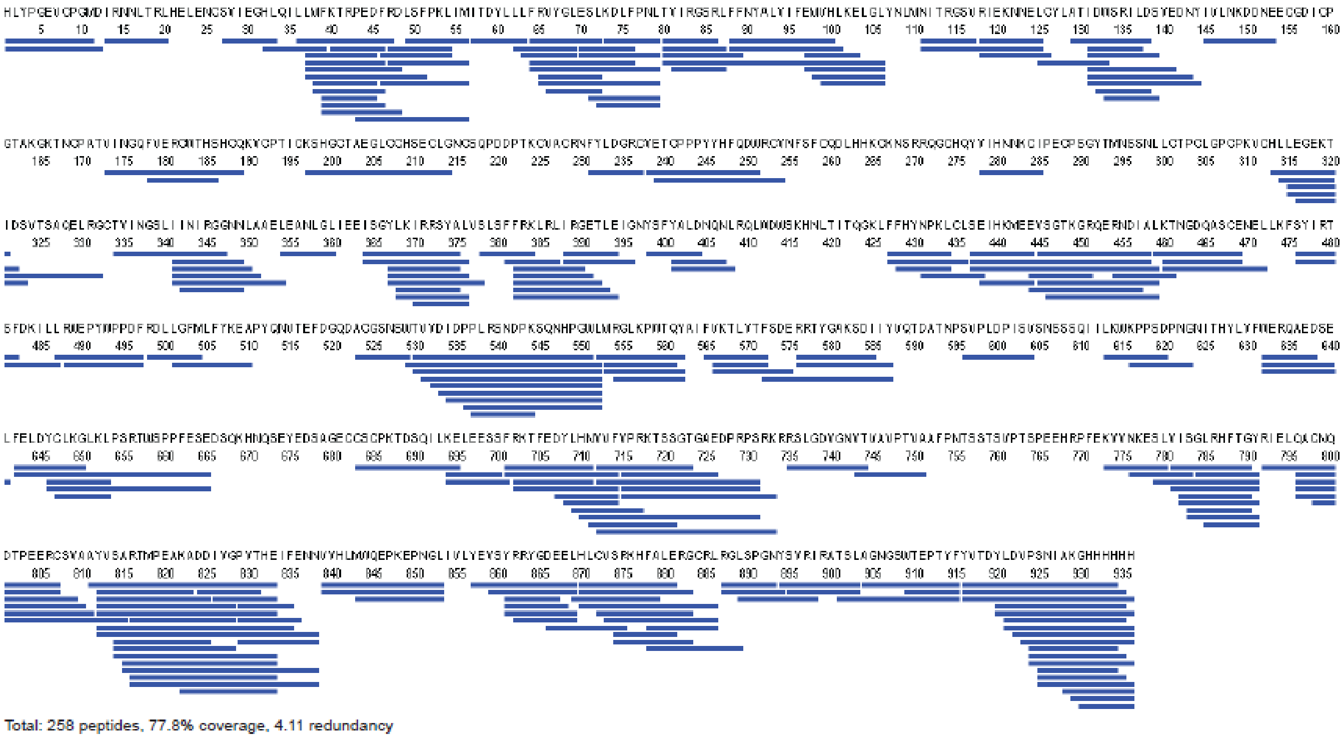

Figure S3

Figure S3. HDX-MS Sequence Coverage Map for Insulin Receptor Ectodomain Complexed with Human Insulin. Sequences underlined in blue were the actual peptic peptides detected in the HDX-MS experiments. A typical sequence coverage was roughly 77%. The number of peptic peptides detected was greater than 250 with more than 4.1 sequence redundancy.

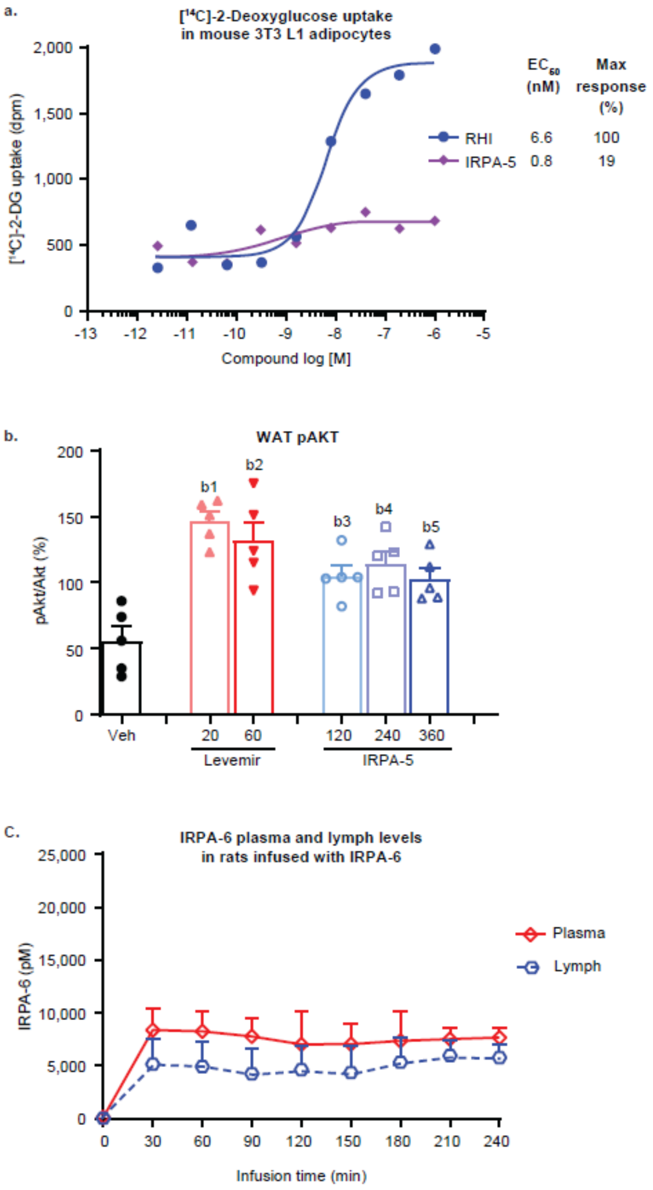

Figure S4

Figure S4. Glucose update in 3T3 L1 adipocytes, IRPA-mediated signaling in mouse adipose tissue and peripheral distribution of IRPA. RHI and IRPA-1 stimulated 2-deoxyglucose uptake in differentiated 3T3 L1 adipocytes was measured (S4a). Mouse white adipose tissue pAkt levels (S4b) were quantified in the same manner as other tissues described in the Legend of Fig. 3c-d. p value (vs. Vehicle) is calculated using one way ANOVA with Dunnett's multiple comparisons test: b1<0.0001, b2<0.0001, b3=0.008, b4=0.002, b5=0.01. n=5 per group. IRPA-6's plasma and lymph levels (n=4; mean ± SEM) during its infusion in cannulated rat (S4c) were measured in the same way as described in the Figure Legend of Fig. 3j-k. There was no significant difference between plasma and lymph IRPA-6 levels using two-way mixed effects ANOVA analysis with Geisser-Greenhouse correction. Source data are provided as a Source Data file.

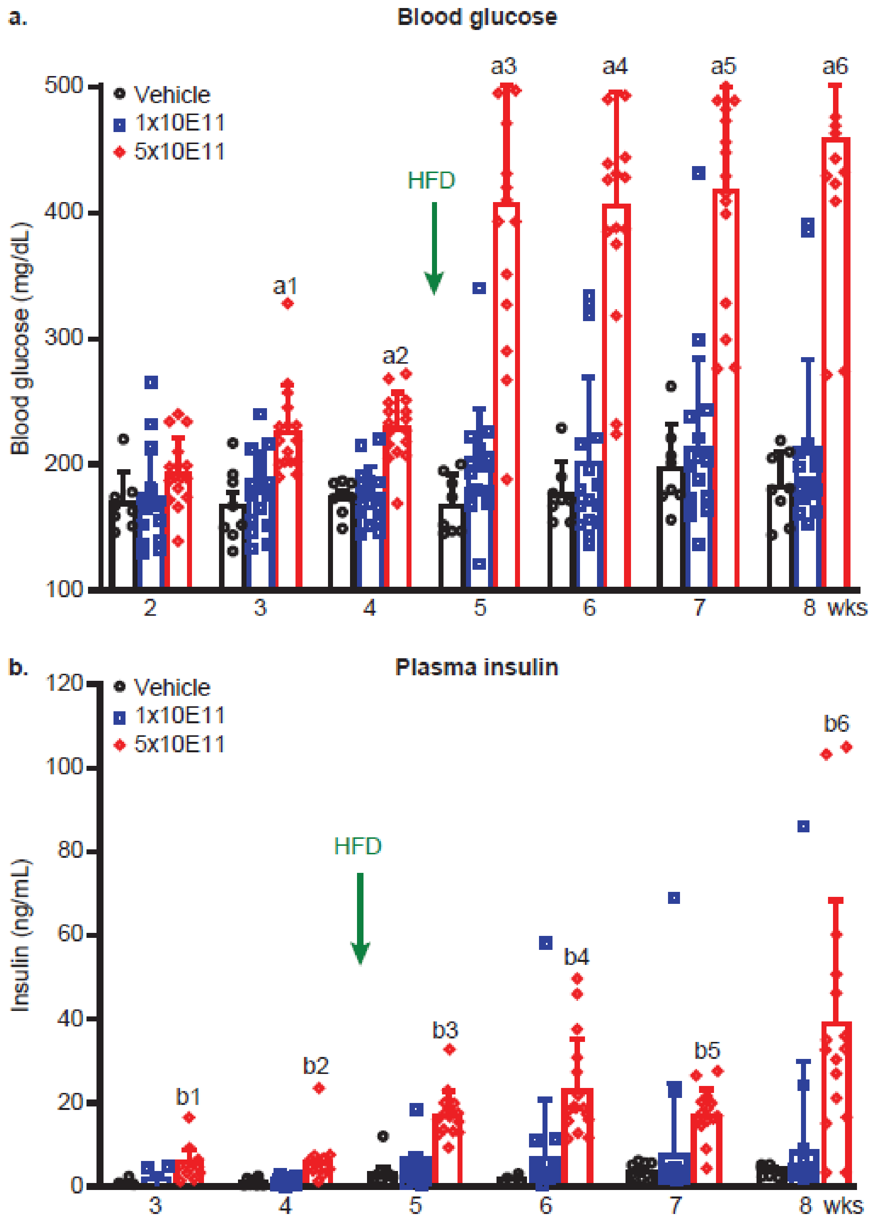

Figure S5

Figure S5. AAV-LIRKO Mouse Model Generation. Blood glucose (S5a) and plasma insulin (S5b) of AAV-cre-mediated LIRKO mouse model before and after HFD treatment. IR cKO mice tail vein injected with two different doses of AAV-cre. Mice were studied 2 weeks following AAV injection (as indicated on x-axis). HFD diet treatment was initiated week 4 after AAV injection. Graphs are mean ± SD. p value (vs. Vehicle) is calculated using one way ANOVA with Dunnett's multiple comparisons test: a1=0.003, a2-a6<0.0001; b1=0.0001, b2=0.004, b3<0.0001, b4=0.0002, b5=0.005, b6=0.002. Sample size for vehicle, 1x10E11 or 5x10E11 group is 8, 16 or 16 respectively. Source data are provided as a Source Data file.

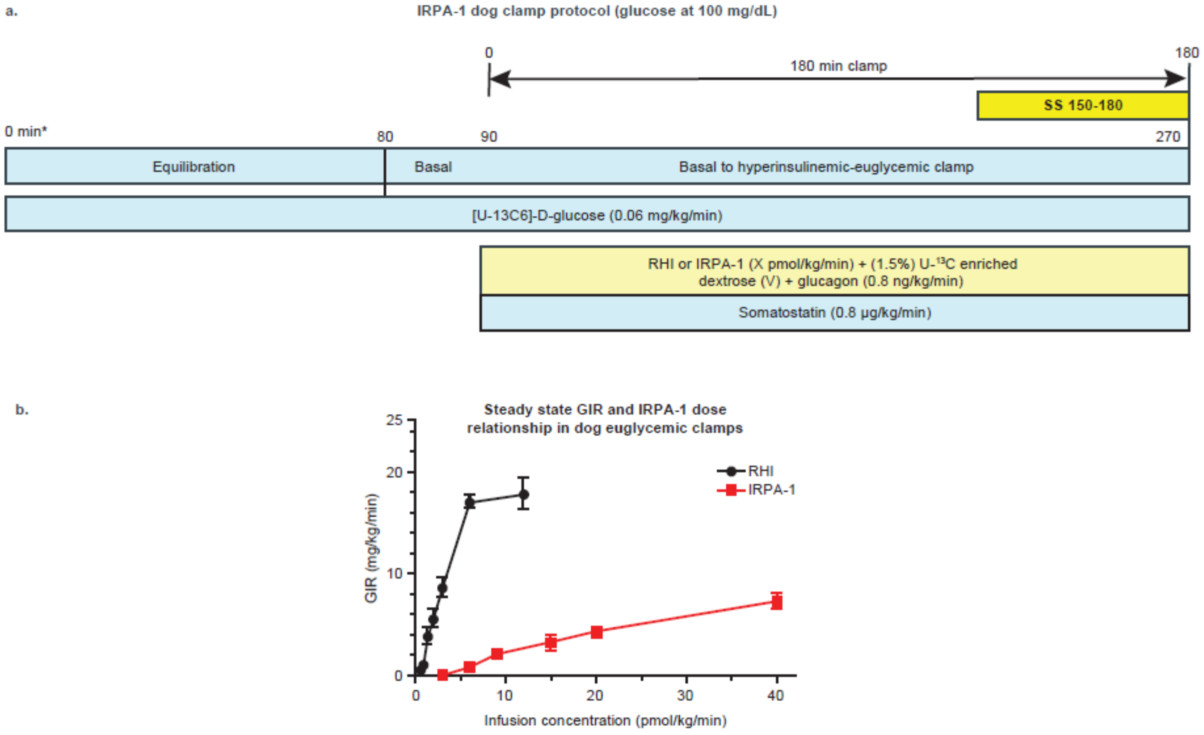

Figure S6

Figure S6. IRPAs Have Hepato-adipose Preferential Action in Higher Species. IRPA-1 has smooth and less steep dose-GIR relationship in dog euglycemic clamp studies (S6a-b). Clamp procedure is described in S4a. Animals were primed with Dextrose (45-46% w/v) enriched with 1.5 % [U-^{13}C6]glucose. Glucose was clamped at 100 mg/dL. Plasma enrichment samples for evaluation of EGP and Rd were collected at 0, 10, 20, 30, 40, 50, 60, 70, 80, 90 (+0 min after clamp) minutes from initiation of the procedure and then taken at +30, +60, +90, +100, +110, +120, +150, +160, +170, +180 minutes during clamp. Blood glucose measurement were performed every 10 min. RHI infusion rates were 0.6, 0.9, 1.4, 2, 3, 6 and 12 pmol/kg/min (pkm). IRPA-1 infusion rates were 1.5, 3, 6, 9, 15, 20 and 40 pkm. All data is presented as mean ± SEM. n=4-8 animals/dose group (IRPA-1), n=3-8 animals/dose group (RHI). Dose group with corresponding sample size in parathesis is RHI at 0.6 (7), 0.9 (8), 1.4 (8), 2 (8), 3 (8), 6 (3), 12 (8) pmol/kg/min; IRPA-1 at 3 (4), 6 (8), 9 (8), 15 (6), 20 (8) and 40 (8) pmol/kg/min. Source data are provided as a Source Data file.

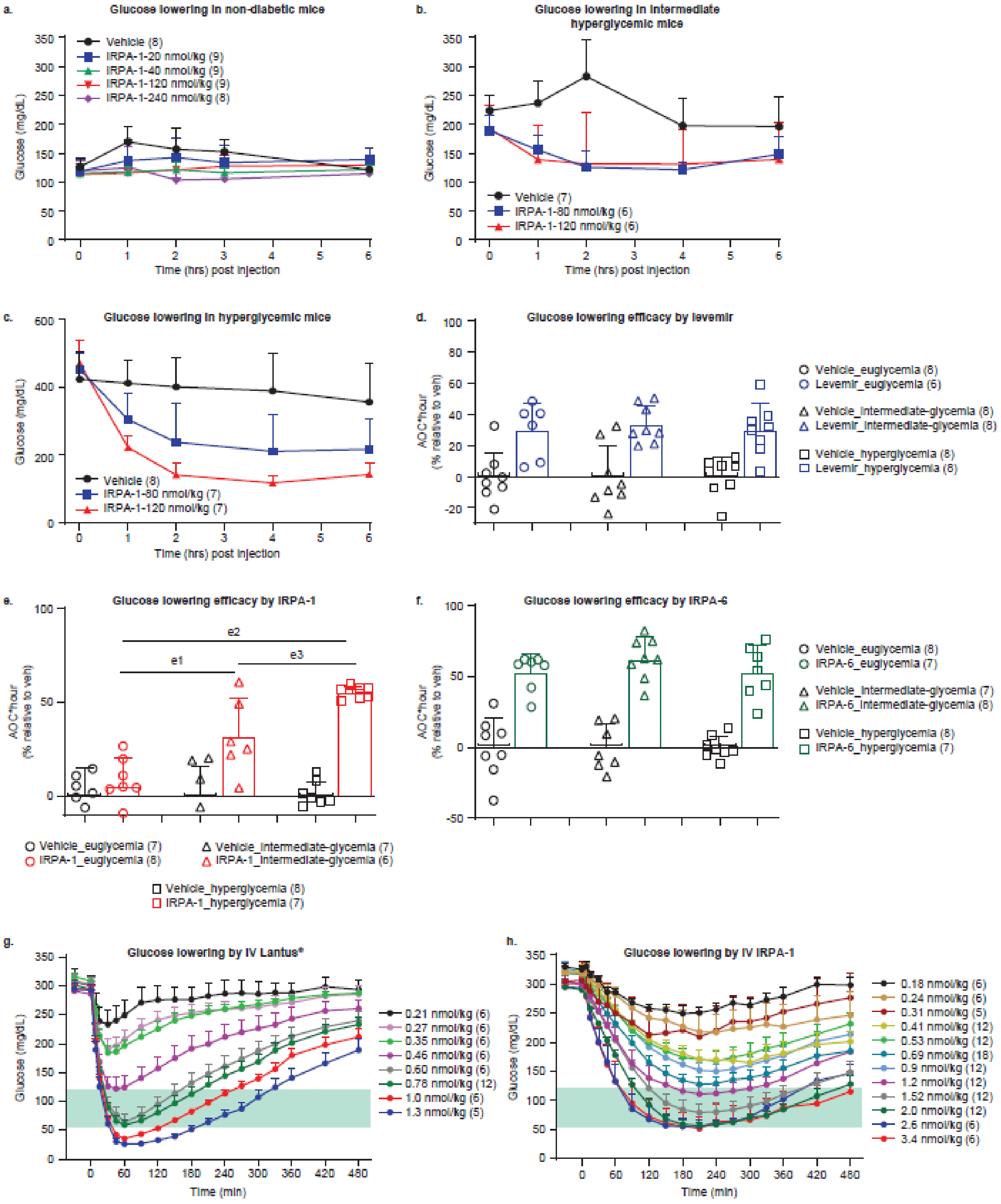

Figure S7

Figure S7. IRPAs are Efficacious in Glucose Lowering with Improved Therapeutic Index. IRPA-1 displays differential glucose lowering efficacy in diabetic vs. non-diabetic mice (S7a-c). IRPA-1 in non-diabetic mice (S7a; baseline glucose range: 100-150 mg/dL), STZ-induced intermediate hyperglycemic mice (S7b; 150-250 mg/dL) or

STZ-induced severe hyperglycemic mice (S7c; 350-500 mg/dL). All data in S7a-c is presented as mean ± SD. N number is indicated on graph (legend in parathesis). Differential glucose lowering potency of insulin dimers is unique to partial agonists (S7d-f). Glucose lowering efficacy is shown for non-diabetic mice (S7d), STZ-induced intermediate hyperglycemic mice (S7e) or STZ-induced hyperglycemic mice (S7f) upon single dose s.c. dosing. Glucose lowering efficacy is expressed as area over the curve (AOC) relative to vehicle-treated group (increase in percentage relative to vehicle group, average of which is set as 0). IRPA-1: dimer partial agonist; IRPA-6: dimer full agonist. Levemir, IRPA-1 or IRPA-6 dose used was 12, 120, 20 nmol/kg, respectively. All treatment effect was normalized to its own vehicle group. N number is indicated on graph (legend in parathesis). Graphs shown are mean ± SD. p value is calculated using one way ANOVA with Tukey's multiple comparisons test: e1=0.02, e2<0.0001, e3=0.04. Corresponding analysis with Levemir or IRPA-7 have p>0.05. Intravenous dose escalation studies in diabetic minipigs (S7g-h; data presented as mean ± SEM). Dose-escalation of Lantus® in fasted diabetic Yucatan minipigs (S7g; 0.21, 0.27, 0.35, 0.46, 0.6, and 1 nmol/kg, n=6; 0.78 nmol/kg, n=12; 1.3 nmol/kg, n=5). Dose-escalation of IRPA-1 in fasted diabetic Yucatan minipigs (S7h; 0.18, 0.24, 2.6 and 3.4 nmol/kg, n=6; 0.31 nmol/kg, n=5; 0.41, 0.53, 0.9, 1.2, 1.52 and 2 nmol/kg, n=12; 0.69 nmol/kg, n=18). Both insulins were administered as a single i.v. bolus, and glucose levels were monitored for 8 hours. Target glucose levels are indicated by green area between 55 and 120 mg/dL. Threshold for hypoglycemia in minipigs is defined at plasma glucose of 55 mg/dL. Source data are provided as a Source Data file.
