# Supplementary material for: Functionally selective signaling and broad metabolic benefits by novel insulin receptor partial agonists
Source: Nat Commun. 2022 Feb 17;13:942. doi: 10.1038/s41467-022-28561-9 (PMC8854621; doi:10.1038/s41467-022-28561-9)
Supplement: Supplementary file 2 — Reporting Summary [file 41467_2022_28561_MOESM2_ESM.pdf]

nature research

Corresponding author(s): Songnian Lin, James Mu

Last updated by author(s): 2021-12-9

Reporting Summary

Nature Research wishes to improve the reproducibility of the work that we publish. This form provides structure for consistency and transparency in reporting. For further information on Nature Research policies, see our Editorial Policies and the Editorial Policy Checklist.

Statistics

For all statistical analyses, confirm that the following items are present in the figure legend, table legend, main text, or Methods section.

n/a	Confirmed	
☐	☒	The exact sample size (n) for each experimental group/condition, given as a discrete number and unit of measurement
☐	☒	A statement on whether measurements were taken from distinct samples or whether the same sample was measured repeatedly
☐	☒	The statistical test(s) used AND whether they are one- or two-sided<br>*Only common tests should be described solely by name; describe more complex techniques in the Methods section.*
☐	☒	A description of all covariates tested
☐	☒	A description of any assumptions or corrections, such as tests of normality and adjustment for multiple comparisons
☒	☐	A full description of the statistical parameters including central tendency (e.g. means) or other basic estimates (e.g. regression coefficient) AND variation (e.g. standard deviation) or associated estimates of uncertainty (e.g. confidence intervals)
☒	☐	For null hypothesis testing, the test statistic (e.g. F, t, r) with confidence intervals, effect sizes, degrees of freedom and P value noted<br>*Give P values as exact values whenever suitable.*
☒	☐	For Bayesian analysis, information on the choice of priors and Markov chain Monte Carlo settings
☒	☐	For hierarchical and complex designs, identification of the appropriate level for tests and full reporting of outcomes
☒	☐	Estimates of effect sizes (e.g. Cohen's d, Pearson's r), indicating how they were calculated

Our web collection on statistics for biologists contains articles on many of the points above.

Software and code

Policy information about availability of computer code

Data collection	Cryo-EM and HDX-MS data were collected by Leginon and MassLynx4.1, respectively.
Data analysis	GraphPad Prism (v7 and up), PLGS 3.0.3 and DynamX 3.0 for HDX analysis, cryoSPARC v0.6.5 and v2.4.6 for cryoEM image analysis

For manuscripts utilizing custom algorithms or software that are central to the research but not yet described in published literature, software must be made available to editors and reviewers. We strongly encourage code deposition in a community repository (e.g. GitHub). See the Nature Research guidelines for submitting code & software for further information.

Data

Policy information about availability of data

All manuscripts must include a data availability statement. This statement should provide the following information, where applicable:

- Accession codes, unique identifiers, or web links for publicly available datasets
- A list of figures that have associated raw data
- A description of any restrictions on data availability

All relevant data in the manuscript and supplementary section are available in Data Source file in Supplementary Information or data bank with access information provided.

Field-specific reporting

Life sciences study design

All studies must disclose on these points even when the disclosure is negative.

Sample size	In vitro studies include at least 2 biological replicates as indicated in Figure Legends. Samples size of each in vivo study is indicated in Figure Legends, with n=3 or higher. Sample sizes were chosen following historical experience that supports these sample sizes are sufficient to detect relevant biological effects.
Data exclusions	No data was excluded, with the exception of a few cases detailed below and indicated in the Data Source file. The general guideline is to exclude individual measurement if it's outside the group mean +/- 3 times standard deviation. Cases of data exclusion are: 1) plasma insulin value of one 240-min sample and Lymph insulin sample of one-30 min sample (Fig. 3j); 2) plasma insulin value of one 90-min sample (Fig. 3k); 3) liver triglyceride of one vehicle sample (Fig. 6d). All these values are clearly outliers from the corresponding group and match our exclusion criteria.
Replication	All experiments were conducted with multiple biological replicates as detailed in Methods and Figure Legends.
Randomization	Randomization of animals were performed, according to glucose, body weight, and additional parameters as needed, in each in vivo study. Animals were allocated into control and experimental groups randomly and, at the same time, ensured every group started with comparable average value of the key parameter(s).
Blinding	With the exception of minipig studies, investigators were not purposely blinded to treatments in most cases. Animals in these studies were completely randomized and allocated evenly across control and treatment groups. In most cases, multiple investigators were involved in the study at different stages, such as animal randomization, dosing and measurements at different time points during the study, which minimized systemic bias during these studies based on institutional experience.

Reporting for specific materials, systems and methods

We require information from authors about some types of materials, experimental systems and methods used in many studies. Here, indicate whether each material, system or method listed is relevant to your study. If you are not sure if a list item applies to your research, read the appropriate section before selecting a response.

Materials & experimental systems

n/a	Involved in the study	
☐	☒	Antibodies
☐	☒	Eukaryotic cell lines
☒	☐	Palaeontology and archaeology
☐	☒	Animals and other organisms
☒	☐	Human research participants
☒	☐	Clinical data
☒	☐	Dual use research of concern

Methods

n/a	Involved in the study	
☒	☐	ChIP-seq
☒	☐	Flow cytometry
☒	☐	MRI-based neuroimaging

Antibodies

Antibodies used	Phospho-protein detection in this manuscript used HTRF and Meso Scale Discovery (MSD)-based commercial cellular kits. Custom MSD IR MSD assays were developed using the following antibodies: pY960 (rabbit mAb, Cell Applications #CB4378); pY1150 antibody (rabbit monoclonal antibody 19H7, Cell Signaling #3024); pY1345 (rabbit mAb 14A4, Cell Signaling #3026); SULFO-tag anti-rabbit antibody (MSD cat# R32AB); rabbit anti-insulin receptor b (C-19, Santa Cruz sc-711). No antibody was used for Western blot in this manuscript.
Validation	All HTRF and MSD kits used were certified by manufacturers and validated in our studies (e.g. robust standard curves demonstrated) using control ligands.

Eukaryotic cell lines

Policy information about cell lines

Cell line source(s)	CHO and HEK293 cells were originally obtained from ATCC and were used to generate stable cell lines expressing target gene of interest, e.g. insulin receptor. These cells have been previously reported, such as JCI Insight. 2018 11; 3(1):e97476 and Diabetes. 2018; 67(2):299-308.
Authentication	All cell lines in relevant studies were maintained by dedicated scientists and their identities were verified regularly, by morphology and cell-specific functional assays.
Mycoplasma contamination	All cells in use were screened on a regular basis for mycoplasma to ensure lack of contamination (negative for mycoplasma contamination).

Commonly misidentified lines (See ICLAC register)	No commonly misidentified cells were used in current studies.

Animals and other organisms

Policy information about studies involving animals; ARRIVE guidelines recommended for reporting animal research

Laboratory animals	Male C57BL/6 mice 8 week and older; male Sprague-Dawley rats 8 week and older; male Yucatan minipigs, 10 months-3 years old; male beagle dogs, 2-8 years old. All animals were housed in a room maintained at constant temperature (23°C ± 2°C) and humidity (55% ± 15%) under controlled conditions of 12-hour light/12-hour dark cycles.
Wild animals	No wild animals were used in the study.
Field-collected samples	No field collected samples were used in the study.
Ethics oversight	All animal procedures were reviewed and approved by the research laboratories of Merck & Co., Inc., Kenilworth, NJ, USA, Institutional Animal Care and Use Committee. In the case of minipig studies, animal procedures were also approved by Sinclair Research Center (Auxvasse, MO, USA) Institutional Animal Care and Use Committees.

Note that full information on the approval of the study protocol must also be provided in the manuscript.
